# Bridging the gap: a cross-cultural examination of PCIT training experiences across Black, White, Asian, and Multiracial clinicians

**DOI:** 10.3389/frcha.2025.1549333

**Published:** 2025-12-08

**Authors:** Felipa T. Chavez, Kaela Farrise Beauvoir, Eyram Agbeli, Sierra Coffey, Emily Aron, Erica E. Coates

**Affiliations:** 1 School of Psychology, Florida Institute of Technology, Melbourne, FL, United States; 2 Department of Counseling, Clinical and School Psychology, University of California-Santa Barbara, Santa Barbara, CA, United States; 3 Department of Psychology, Georgetown University, Washington, DC, United States; 4 Department of Pharmacology & Physiology, Georgetown University, Washington, DC, United States; 5 Department of Psychiatry, Georgetown University Medical Center, Washington, DC, United States

**Keywords:** parent child Interaction therapy (PCIT), evidence-based programs (EBP), cultural sensitivity, clinical training, Black families, racial differences

## Abstract

**Introduction:**

Parent-Child Interaction Therapy (PCIT) is a strongly evidence-based treatment (EBT) for disruptive behaviors in young children. However, PCIT research with Black families has identified notable disparities particularly with regard to more frequent and earlier attrition from treatment and disparate outcomes. Prominent etiological explanations lie in PCIT's perception as a Eurocentric treatment embedded within discriminatory systems and, therefore, unable to meet Black families' unique cultural needs. The present study sought to better understand the training experiences of PCIT clinicians broadly, and with a specific eye towards illuminating the cultural congruence and incongruence of PCIT training towards the goal of serving Black families.

**Methods:**

A racially diverse (Black *n* = 10; White *n* = 8; Asian *n* = 2; Multiracial *n* = 2) sample of PCIT clinicians (*n* = 22) was interviewed using a structured interview protocol. Transcripts from the virtual interviews were analyzed by a 4-person coding team using thematic analysis with both inductive and deductive code development. Clinicians were also administered a modified measure of self-perceived provider cultural competence. Independent samples t-tests were performed to compare perceptions of cultural competence among various racial groupings.

**Results:**

Several themes were identified including a corroboration by clinicians across racial groups regarding perceptions of high-quality but very White-normed training experiences, the need for more Black PCIT clinicians, and inadequate preparation for tailoring PCIT towards Black families. Additionally, while other research suggests that Black clinicians feel adept at culturally interpreting certain PCIT language and larger concepts they deemed inappropriate for servicing Black families, current findings suggest that both White and Asian American clinicians reported less confidence in knowing how to address the unique needs of Black families with PCIT. Quantitatively, significant differences were found in the level of perceived cultural competence between White and non-White clinicians.

**Discussion:**

Overall, this study highlights areas for growth in PCIT training and the development of a diverse body of PCIT clinicians able to meet the needs of Black families. Implications for clinical training development and implementation, as well as clinician recruitment and retention, are discussed.

## 1 Introduction

### 1.1 Background: PCIT with Black families

Black children are disproportionally over-diagnosed with disruptive behavior disorders (1, 2). *Parent Child Interaction Therap*y [PCIT; (3)] is considered the staple treatment for families with young children exhibiting these disruptive behaviors and other non-compliance issues (4, 5). PCIT is an evidence-based treatment (EBT) program that has demonstrated tremendous efficacy in improving disruptive behaviors, even when compared to other mental health treatment modalities (5). PCIT treatment is conducted in two phases: *Child Directed Interaction* (CDI), and *Parent Directed Interaction* (PDI). The CDI phase focuses on fostering appropriate child behaviors through caregivers' use of prosocial verbalizations and positive behaviors during play interactions where the caregivers are coached to follow the child's lead (3). Caregivers' skill acquisition is represented by the “PRIDE” acronym representing caregivers' ability to *praise, reflect, imitate*, and *describe* their children's positive behaviors, while demonstrating great *enjoyment* and *enthusiasm* during caregiver-child play interactions. Caregivers' acquisition of the requisite PRIDE skills in CDI is subsequently followed by the second phase of treatment, PDI. During PDI, caregivers are instructed in appropriate command-giving that best fosters appropriate expectations and elicits compliance, along with contingencies for non-compliance in the form of a failsafe timeout procedure.

Across modalities, research in EBTs are fraught with participant samples that are not racially inclusive in part due to poor retention rates of minoritized individuals and families (6–8). To that end, there remains considerable questioning regarding PCIT's accessibility and evidence-base for Black families (4, 9). In addition to a general dearth of Black participants in PCIT research, as evidenced by just a handful of studies having majority Black samples (9), retention issues are challenging. In PCIT treatment, Black families drop out of treatment at a rate of 56% (7), compared to their White counterparts at 36% (10). Further, in a group-based adaptation of PCIT, Blair et al. (11) found that attrition was significantly associated with race, wherein Black caregivers had a 6.12 higher likelihood of dropout, compared to other races. Another study of PCIT showed that having a Black caregiver was significantly associated with child attrition during the first phase of treatment (12). Accordingly, attrition for Black families is seen earlier than with families of other races, including as early as intake (13), and is significantly associated with caregivers being Black in multiple PCIT studies (11, 12). Some potential etiological explanations for the high attrition lie in the perceptions Black families may hold regarding PCIT's evidence-base being normed on White families, thereby making the treatment less culturally credible (4, 14, 15). In interviewing Black PCIT clinicians about their experiences working with Black families, Coates et al. (15) found that clinicians consistently noted PCIT is: (1) perceived by Black families as a White treatment for White families, (2) embedded within and aligned with discriminatory systems, and (3) unable to meet Black families' unique cultural needs. As a result of the elevated early attrition rates among Black families, they are less likely to advance through the two major phases of PCIT treatment, and thereby are unlikely to reap the full therapeutic benefits.

Despite some evidence that PCIT is efficacious for Black families who complete treatment (16), substantiated research has been limited, and mirrors the larger unmet mental health need of Black youth in the United States, as well as the well-documented health disparity between people of color (POC) and their White counterparts (17–19). Various solutions have been proposed to address the disparities in mental health access and acceptability between POCs and their White counterparts. These solutions include: (1) requiring training in cultural competence, humility and sensitivity, and (2) increasing efforts and resources focused on racially/ethnically diversifying the licensed mental health workforce. In addition, recommendations have been made to employ lay health workers of similar racial/ethnic backgrounds to meet the unmet need for services. Both strategies for diversifying the mental health workforce aim to ensure greater buy-in among Black families for treatments that present as more Eurocentric in nature (20–23).

### 1.2 EBT cultural competency trainings for clinicians

Related to the first proposed solution for requiring trainings to foster cultural competency, several studies have shown the enhanced benefit of providers' cultural competence in decreasing rates of early termination that plague many EBTs, such as PCIT, and improving client outcomes [(24); see (25) for a meta-analytic review]. *Cultural competence* is defined as the ability to effectively collaborate and treat clients from diverse backgrounds based on one's ability to understand and respect the values, attitudes, beliefs, and cultural rituals that shape behaviors across diverse groups (26). Accordingly cultural competency is assessed across the three dimensions of: (1) *awareness* and understanding of the importance and influential nature of cultural context on identity, relationships, and life processes, (2) possessing specific cultural *knowledge* relative to various cultural groups at the multiple layers of diversity, identity, and intersectionality*,* 3) and finally being able to apply such awareness and knowledge to *skills application* (27). With respect to the *skills application* domain, clinicians must be receiving training in how to implement culturally attuned tailoring of specialized EBTs, such as PCIT, which require certification. To that end, there have been demonstrated efforts to bring PCIT to the international stage of serving families across the world, through the manual's multiple translations in various languages (e.g., Spanish, French, Japanese, Mandarin, German, Korean, and Dutch to name a few). More recently, there have been strides in offering clinicians guidance on broad cultural tailoring of PCIT treatment through a new paradigm called *MY PCIT* (28, 29).

However, to date, with the exception of one pilot (30), there have been little to no established research regarding standardizing a culturally adapted PCIT *training model* for certifying clinicians, that would offer cultural competency training in the specific elements of *awareness, knowledge*, and culturally-tailored *applications* for addressing the unique needs of Black families within the United States. Nor has there been any research indicating clinicians' levels of cultural competence in the dissemination of PCIT to Black families after receiving PCIT training and certification. Doing so is paramount when one considers Black families' elevated rates of early attrition in PCIT, thereby contributing to some of the mental health treatment disparities facing Black communities. Therefore, ameliorating such disparities must start with training clinicians to be culturally competent at tailoring PCIT to the specific needs of Black families. Trainings in EBTs, including PCIT, should incorporate instructional training that highlights core cultural sensitivity elements of awareness, knowledge, and even more importantly the specific tailoring application of PCIT skills to the unique Black parenting experience.

### 1.3 Black caregivers' unique parenting needs for *racial socialization* practices

Black families face unique sets of marginalizing experiences within discriminatory systems, that place the lives of their Black youth at greater risk. As such, Black caregivers have developed cultural ethno-theoretical parenting strategies (31) that unfold in very specific needs during their *racial socialization* practices in raising, and preserving the lives of their Black youth (32–35). For example, the infamous rite of passage “*The Talk*” entails preparing Black children for how to conduct themselves when interacting with public authorities (36, 37). The purposes of which is to increase the likelihood of their survival and return home. Addedly, the promotion of *Black Pride* serves to bolster against the negative stigmatizing effects of internalized racism in the cultivation of a healthy racial identity, self-image, and self-esteem, leading to positive mental health outcomes (36, 38–42). While “*The Talk,*” focuses on protecting their Black children, it does tend to increase children's anxieties and can serve as a subsequent trigger for transgenerational discrimination trauma, which must be managed, if and when it comes up in the therapeutic context. The bolstering of *Black Pride* in the promotion of healthy *Racial Identity* development (43), are equally crucial cultural elements of Black racial socialization parenting. PCIT possesses core elements and skills, which are well aligned to both of these key racial socialization parenting goals. If culturally tailored, PCIT is well poised for supporting Black caregivers in their ethno-theoretical parenting goals for both the life preservation and flourishing of their Black youth (44–46). PCIT's proven efficacy with managing childhood anxieties (47), healing trauma symptomatology (48), and the promotion of positive self-esteem and ego strength, with reciprocal benefits for caregivers (49, 50), are all integral elements for racial socializing parenting.

However, clinicians must first receive such culturally competent instruction in tailoring PCIT for Black parenting through their clinician EBT trainings, to ultimately be effective ambassadors of PCIT. Through successful training in such highly tailored cultural competencies for working with Black families, successful culturally trained clinicians will be well positioned to highlight PCIT's relevancy for their Black families. Doing so, may ultimately secure Black families' trust in PCIT, and potentially reduce the recidivism rates witnessed. However, these gaps in examining the cultural competencies of PCIT clinician trainings, begs the question whether clinicians' feel adequately trained in the application and tailoring of PCIT for working with their Black families. If not, this lack of cultural competence may contribute to the higher rates of attrition experienced among Black families in PCIT. As Black families may potentially feel PCIT does not sufficiently address their cultural needs based on their lived experiences.

### 1.4 Ethnic/racial concordance in EBTs

Related to the second solution offered for addressing mental health treatment disparities, the low numbers of under-represented providers of color suggest a great need for more POCs to be trained in EBTs. Currently, only 9% of psychologists identify as either Black or Latine (51), while these two racial groups combined, make up an estimated 33% of the U.S. population (52). Addedly, not only is there a general dearth of mental health POC providers, but there is also a substantial underrepresentation of these groups within community mental health settings where the majority of clients of color are seen (53). Additionally, many argue that the underrepresentation of POC providers in clinical mental health fields represents a clogged clinical training pipeline*,* especially for those trained to work with children and adolescents (54). More specifically, an estimated 80% of community-based PCIT clinicians identify as White [e.g., (55)]. Thus, these statistics suggests that certifying organizations should strive to increase the number of trained POC providers, who can disseminate treatment for ethnically and racially diverse clientele with the hopes of improving treatment outcomes.

Increasing the number of certified POC providers has many possible benefits including better representation among decision makers and developers of EBTs, as well as increased access to under-reached communities who may be understandably distrustful of mental health services and providers (56, 57). Such mistrust stems from a long history of racism and prejudice in psychology, and discriminatory practices within healthcare (21, 58). Relatedly, one study of community mental health clinicians found that POC clinicians were better at adapting EBTs to meet the needs of clients of color than White clinicians (59). In another study, attrition was found to be higher specifically among Black clients with White providers (60). Specific to Black families in PCIT treatment, studies have highlighted the perception that some Black families see PCIT as a treatment geared towards White families (14, 15). Thus, the lack of Black providers is a key consideration for Black families who struggle to access quality mental health services by trusted PCIT-trained professionals emanating from their communities. Such perceptions regarding the lack diversity within the PCIT community's mental health workforce, is something not to be ignored, and behooves research to more closely assess among their PCIT trained clinicians. Doing so will subsequently inform next steps for greater inclusivity in training spaces and providers.

#### 1.4.1 Ethnic/racial provider concordance in EBTs for Black families

In addition to a broad call for a more ethnically and racially diverse mental health workforce, researchers, clinicians, and consumer groups have offered racial matching as a specific strategy for improving care, and mitigating the treatment accessibility challenges that exist for Black communities. For example, when asked for feedback on tailoring EBTs to Black communities, an advisory council made up of Black and Latine caregivers with young children, identified ethnically/racially concordant providers, instructional tools, and resources as important requirements (61). Further, while the research on the efficacy of racial concordance related to outcomes in healthcare is mixed for most racial groups (62, 63), the findings regarding Black clients have been more conclusively in favor of racial provider concordance. Many Black clients have a preference for a racially concordant providers, and those who do, tend to feel more comfortable in treatment, and are more willing to stay in treatment (64). Thereby, reducing attrition and improving the therapeutic working alliance, which are crucial to effective treatment (63–65). In a metanalytic review, Cabral and Smith (63) found that Black clients tended to rate Black providers more favorably and express a significant preference for Black providers when seeking treatment. In another study, this preference for Black providers was shown to be especially strong for Black people with higher levels of race centrality in their identities (42, 64). Such findings point to the benefits of increasing certified Black clinicians who reflect the ethnic/racial communities they serve in order to improve treatment retention. This is particularly relevant for Black families in PCIT, where early attrition is prevalent, and families stay in treatment longer before seeing the same improvement in outcomes, as compared to their White counterparts (7, 11, 12, 66). Thus, the potential benefits of provider racial concordance for Black families in PCIT cannot be ignored. Potential benefits of racial/ethnic provider concordance include increased buy-in so Black families may reap the empirically-proven benefits of PCITs in managing children's behaviors. In fact, one study of Black clinicians who were trained in standard PCIT reported independently taking the initiative to culturally tailor their interventions with Black families, based on their cultural insider knowledge about their community to better serve their Black clients (15). Therefore, racial/ethnic matching in PCIT with respect to Black families, could serve to bring greater understanding of life circumstances, based on shared lived experiences (67). Thus, validating Black Families' perceptions of being seen and understood, has the hopes of improving retention rates. All of which informs future directions for the recruitment and accessibility processes for increasing the number of Black PCIT trained providers.

#### 1.4.2 The link between ethnic/racial concordance and cultural competence

That being said, it is equally plausible that these positive effects of concordant provider preferences witnessed among Black clientele, may in fact be an indicator of cultural competence as a function of shared life experiences. That is, *cultural competence* may in fact serve as the more significant predictor of treatment outcomes. **S**tudies have demonstrated that POC providers tended to score higher on provider cultural competency (PCC) standardize measures (68). Addedly, in a recent study on the effects of race matching, researchers found that the providers' cultural competence was more predictive of treatment outcomes (68, 69). Thus, there is the potential that when Black clients show higher preference for race concordant providers (63, 64), that the provider being Black served as an indicator of *cultural competence* due to the perception of the provider's shared lived experience in discriminatory systems. Therefore, signaling that the more salient etiological factor to promoting success with Black families in PCIT, is having culturally competent PCIT providers, regardless of race. Thus, having culturally tailored PCIT training standards that better aligned with the values and cultural traditions of Black families may be necessary for improving treatment retention and outcomes, regardless of the race of the clinician. However, little is currently known about the cultural competency training experiences of PCIT clinicians more generally, and more specifically for providers of color seeking PCIT certification who may have different needs in their PCIT training goals if they already possess insider knowledge about Black communities (15, 30). Hence, it is important to assess clinicians' appraisal of their PCIT-training, and its efficacy in building clinicians' cultural competence in working with Black families.

### 1.5 PCIT training

Thus far, what we do know about PCIT clinician training is as follows. Certification requirements for PCIT-certified clinicians were first established in 2009 (70) with the most recent revision occurring in 2018 (71). Basic training for PCIT certification requires meeting several benchmarks for graduate level clinicians including (1) 40 h of training, (2) ongoing consultation with a certified PCIT trainer until two cases are fully completed, and (3) the passing of a skills-focused test. However, the literature currently does not document a standardized protocol for PCIT clinician trainings regarding *how* it is to universally be conducted, or what information is to be transmitted during one's training experience (72). Therefore, there is nothing referencing the mandatory inclusion of a cultural competency curriculum. The only exception to date, is a small pilot that used a concordant trainer-trainee PCIT training model for Black and Latine clinicians servicing Black and Latine families of autistic youth (30). This qualitative study highlighted positive appraisals by POC clinicians for the trainer concordant model and culturally adapted curriculum that provided specialized training and instruction in tailoring PCIT to address the specific cultural needs of children with intersectional identities of race/ethnicity and disabilities. However, the small sample size and specialized POC sample, sets limits on the generalizability of the findings. This is especially the case regarding cultural competency training appraisals by White clinicians' with respect to PCIT, and their sense of readiness for working with Black families.

Furthermore, research on PCIT training has shown that clinicians' attitudes towards PCIT impact their skill acquisition and likelihood of eventual certification status. Specifically, in a study of clinicians' perceptions of PCIT training, researchers found that clinicians who dropped out of PCIT training before certification, were more likely to report negative training experiences (73). Identified barriers included the cost of training to their agencies and associated financial burdens with PCIT implementation, difficulty securing a sufficient caseload, and dissatisfaction with the group consultation format, which clinicians did not feel was sufficient for addressing their training and client needs. However, similar to caregiver samples in PCIT research, most previous studies exploring the experiences of clinicians being trained in PCIT included majority White clinician samples (when the race of the clinicians was reported; 73–75). Now while the focus of clinician feedback was not on training experiences, certified Black PCIT clinicians serving Black families have identified continued barriers within the PCIT protocol (15, 30), indicating that clinicians of color serving Black clients may have different perceptions of being trained in and utilizing the PCIT protocol, subsequently impacting their motivations towards PCIT training and certification. Therefore, further research should be done to explore, compare, and contrast the training experiences of racially diverse PCIT therapists to inform training implementation, and more specifically their appraisal of cultural competencies within the context of PCIT.

### 1.6 Current study

It is important that both clients and clinicians of color see themselves and their communities represented within PCIT. This representation is essential for furthering the utility of PCIT as a treatment of choice when working with Black families, whose children have behavioral difficulties, and for which the societal consequences can be more deleterious than their White counterparts (76). However, the perspectives of clinicians serving Black families in PCIT has been minimally explored (15), and not at all explored with a racially diverse group of clinicians nor with respect to their experiences of being trained to be certified PCIT clinicians, and whether those experiences developed, increased, or aligned with their cultural competence. Underdeveloped cultural competence via insufficient cultural connection of PCIT to relevant cultural and societal issues facing Black families can exacerbate implementation, acceptability, and accessibility within Black communities. Thus, a gap in the PCIT literature exists as to racially diverse clinicians' appraisals of their PCIT training experiences in adequately preparing them to provide culturally appropriate PCIT services to their Black families. Given the importance of cultural competence in psychotherapy, clinicians must all be equipped to attend to relevant cultural considerations to improve both client retention and therapeutic outcomes (25). The current study sought to advance the conversation on culturally competent clinical training, specifically for those who have undergone PCIT training and certification. Here, researchers sought to understand the experiences of PCIT clinicians of color, as compared to their non-Black counterparts, with a specific focus on their observations of cultural competence, or lack thereof during their PCIT training. In undergoing this research project, it is the hope that clinicians' perceptions of their training can inform PCIT training for all clinicians working with Black families, by giving insight into what clinicians think is missing in creating a more culturally competent clinical community within PCIT clinician trainings, as well as their perceptions of their own cultural competence. To elicit this data, researchers sought to answer the following questions:

1. How do Black, White, Asian, and Multiracial PCIT clinicians experience their training with a specific lens of cultural sensitivity?
2. How do the experiences of Black PCIT clinicians differ from other clinicians of color? and,
3. How do the training experiences of Black PCIT clinicians differ from White clinicians?

## 2 Methods

### 2.1 Participants

Participants were PCIT clinicians recruited via emailed flyer dissemination through the PCIT International listserv, with one additional clinician referred by another clinician. In order to be eligible to participate in the study, participants were required to: (1) be trained in PCIT or in the process of being trained, and (2) have provided PCIT to one or more Black families. In total, 22 clinicians were interviewed for the study with the sample ranging in age from 28–62 (*Mage* = 38.5, *SD* = 9.43) with ∼95% of participants being women (*n* = 21). Participants identified racially as 45% Black or African American (*n* = 10), 36% White (*n* = 8), 9% Asian or Asian American (*n* = 2), and 9% Multiracial or multiracial (*n* = 2). In the sample, 19 clinicians were fully licensed (86%), with approximately 45% (*n* = 10) holding a doctorate degree, while 55% (*n* = 12) had master's degrees. Among participants, annual income ranged from $24,000 to $400,000 (*M* = $133,227.3, *SD* = $99,177.06). Clinicians had been practicing therapy generally between 4 and 40 years (*M* = 11.93, *SD* = 8.9). Further, clinicians had a range of 1.5 and 35 years providing PCIT (*M* = 6.5, *SD* = 6.94) with estimates of treating between 4 and 350 families (*M* = 60, *SD* = 90), using PCIT. Specific to treatment with Black families, participants' estimates ranged from working with 2 Black families to 280 Black families (*M* = 26, *SD* = ∼58) (See Table 1 for a full summary of participant characteristics).

**Table 1:** Clinicians' demographic characteristics.

Characteristic	Total Sample		Black/African American		White		Asian/Asian American		Biracial/Multiracial	
	*n*	%	*n*	%	*n*	%	*n*	%	*n*	%
Race			10	45	8	36	2	9	2	9
Sex										
Female	21	95	10	100	7	87.5	2	100	2	100
Male	1	5	0	0	1	12.5	0	0	0	0
Education										
Master's	12	55	5	50	5	62.5	2	100	0	0
Doctorate	10	45	5	50	3	37.5	0	0	2	100
Clinical Licensure Status										
Licensed	19	86	9	90	8	100	1	50	1	50
Unlicensed	3	14	1	10	0	0	1	50	1	50
Characteristic	***M***	***SD***	***M***	***SD***	***M***	***SD***	***M***	***SD***	***M***	***SD***
Age	38.52	9.43	38.23	9.39	42	10.23	34	8.49	30.5	0.71
Income	$133,227	$99,177	$124,800	$71,639	$159,875	$126,322	$27,000	$4,243	$175,000	$127,279
Clinical Experience										
Therapy Experience (years)	11.93	8.9	12.5	10.14	13.125	9.35	9	7.07	7.25	0.35
PCIT Experience (years)	6.5	6.94	5.25	3.25	8.67	10.74	5.75	6.01	4.75	3.18
# of PCIT Families Seen	60	90	31.3	25.61	107.38	136.65	55	63.64	20	14.14
# of Black PCIT Families Seen	26.27	58.7	14.7	13.39	51.13	94.65	6.5	4.95	4.5	3.54

*n*, number of participants; *M*, average.

For the purposes of assessing potential racial group differences across the demographic variables, independent t-tests were conducted using R. Given that the sample size was relatively small, participants were aggregated in one of two ways for assessing racial group differences. That is, racial group differences between Black (*n* = 10) and Non-Black clinicians (*n* = 12), as well as Black (*n* = 10) and White Clinicians (*n* = 8) were analyzed, using independent samples t-tests. No significant differences were found between any groups on any of the demographic variables measured (i.e., income, age, years of therapy experience, years of PCIT experience, number of PCIT families seen overall, and number of Black PCIT families seen). We also evaluated differences between master's degree holders and doctorate degree holders in relation to income, age, and therapy experience questions. *T*-tests revealed that on average, there was a significant difference in income [*t*(20) = 2.3136, *p* = .031] between master's degree holders (*M* = 92,583.33, *SD* = 61,227.53) and doctorate degree holders (*M* = 182,000.00, *SD* = 116,289.87).

### 2.2 Procedures

Following flyer dissemination via the PCIT International Association (PCIT-IA) listserv, interested participants contacted the study recruitment team and subsequently screened for eligibility. To establish eligibility and aid in participant selection, the research team emailed potential participants three screening questions (i.e., “How long have you been a practicing PCIT therapist?”, “How many Black families have you used PCIT with in the past year?” and “Approximately how many total Black families have you used PCIT with?”). All clinicians who contacted the study team were eligible. Once eligibility had been established, participants were scheduled for a virtual structured interview, which lasted approximately one hour or less with a designated interviewer. Interviews were scheduled and completed based on the order that clinicians responded with their availability, with preference given to clinicians who reported working with Black families within the past year. Subsequently, four clinicians completed the screening questions that were not ultimately interviewed as they were deemed ineligible from study participation given the amount of time passed since providing PCIT to a Black family. Upon completion of the interview, participants were compensated $100.00 for their time in the form of e-tango gift cards.

Clinician interviews were recorded and transcribed via a HIPPA compliant virtual platform, then transferred to a HIPAA compliant cloud storage platform. Videos were reviewed to ensure recording integrity, and the associated transcriptions cleaned for accuracy with audio recordings.

#### 2.2.1 Interviewers

Interviewers were four Black female students, one at the graduate level, and the other three at the undergraduate level. All interviewers were trained in conducting the structured interview. Interviewers had limited prior knowledge of PCIT, thereby serving as blind interviewers.

### 2.3 Measures

#### 2.3.1 Interview questions

Clinicians were specifically interviewed regarding their experiences in working with Black families in PCIT treatment. The structured interview was designed by the research team in tandem with a consultant experienced in EBT programs' cultural adaptation. The final structured interview was theory-driven regarding previous PCIT cultural adaptations and treatment barriers for Black families, as well as based on the components of PCIT: assessment, *Child Directed Interaction* (CDI), and *Parent Directed Interaction* (PDI). The structured interview contained several sections with respect to clinician demographic information (as described above in the participants section), and with specific probing questions relevant to PCIT's helpfulness to Black families, attrition and perceived contributing factors, recommendations for improvements, clinicians' utilization of culture into treatment, and their general PCIT training experiences, as well as training specific to addressing issues of culture within treatment. For the current study, researchers analyzed the subset of questions related to clinicians' training experiences (see Table 2 for interview questions).

**Table 2:** Interview questions.

Interview questions
How satisfied are you with your PCIT training and/or experience? How did you feel as a Black/White/Asian/Multi-Racial trainee in PCIT? Were there areas that you had questions about or that made you challenge the model in terms of cultural sensitivity? How comfortable do you feel providing PCIT to Black families?

#### 2.3.2 Cultural competence

In addition to answering interview questions, participants completed a 20-item self-report measure of provider cultural competence (PCC), using an adapted version of the Self-Rated Cultural Competence Instrument for Primary Care Providers (68). The instrument is a theoretically derived measure grounded in a variety of published conceptual models representing components of healthcare provider cultural competencies, which include the following domains: Concept of Culture, Relevance of Sociocultural Context, Disparities in Health, Diverse Beliefs and Behaviors, Cross-Cultural Care, and Patient-Centered Communication. It is one of few known provider-level self-report measures of provider cultural competency (PCC) within the specific context of treatment with patients. Participants are instructed to rate their level of agreement to each item using a 6-point Likert scale from 1 (Strongly Disagree) to 6 (Strongly Agree). Across several of the PCC domains, which are reflective of the instrument's subscales, certain items are reversed scored so that when calculating the mean scores, higher scores are indicative of greater levels of endorsement of the subscale attribute. Also, because the instrument was originally designed to use with physicians, items were specifically tailored to be appropriate for mental health clinicians in the current study (see Supplementary Appendix B).

Most importantly, the measure emphasizes the underlying construct of the interpersonal dimensions of cultural competency (CC) between the provider and their patient. Accordingly, Saha et al. (68) believe the “relationship-centered” focus of provider cultural competency (PCC) is the active ingredient for mitigating healthcare disparities among minorities, and more specifically for Black clientele, with respect to treatment adherence and positive outcomes. Saha et al. (68) further cite a number of corroborating findings as lending support for the construct validity of the measure, which include: 1) providers with higher PCC scores experienced more positive treatment outcomes such as, medication adherence, medical self-efficacy, and positive responsiveness to treatment, which reduced racial disparities between their Black and White clients, 2) low scoring PCC providers having worse outcomes with Black clientele exacerbating the disparities in treatment compliance and outcomes, as compared to their White counterparts, and 3) higher PCC scores being among providers of color. In addition, the measure was found to have adequate internal consistency [Cronbach's *α* = 0.76; (68)] for a measure that assesses six domains of PCC as depicted in its six subscales.

### 2.4 Coding strategy and data analytic plan

#### 2.4.1 Theoretical model and guiding tenets

In developing a coding strategy and a comprehensive data analytic plan, multiple methodologies were utilized. In exploring complex social phenomena where existing theories are limited, burgeoning research may employ a grounded theoretical model (77) in tandem with a thematic analysis approach (78). The grounded theoretical model proposes a bottom-up procedure whereby theory is developed based on the emerging themes generated from participant data collection. Thus, a phenomenological approach (78) was interjected with the aim of illuminating and understanding the narratives of participants' lived experiences, rather than starting with a preconceived theory. The next phase of the qualitative analysis involved a socio-constructivist theoretical approach (79, 80) in which the identified emerging themes were analyzed in a collaborative process between the participants' data, and the researchers' interpretation based on shared cultural lenses and perspectives as PCIT clinicians and persons of color. After emerging themes of participants' lived experiences were identified in a data-driven, bottom-up process, then a quantitative thematic analysis was undertaken, whereby identified codes were analyzed for patterns across the entire dataset utilizing count scores, searching for commonalities among participant groupings to create themes (81).

#### 2.4.2 Detailed qualitative analytic outline

In the first phase of qualitative analysis, a *socio-constructivist* theoretical model (79, 80) was employed prioritizing critical data that amplified expressed perspectives and experiences of our Black, Asian, and Multiracial Clinicians (in juxtaposition to their White Clinician counterparts), regarding PCIT training, and PCIT's application in working with Black families. Such approaches are inherently essential when attempting to fill the void of diverse representations in PCIT translational research, especially as it pertains to Black voices, which are frequently omitted from the evidence-based literature for a myriad of reasons.

Second, an initial bottom-up, inductive approach was used to generate a codebook-oriented thematic analytical strategy for evaluating the qualitative structure of the data (82, 83). Using this bottom-up inductive strategy, more specifically outlined by McAlister et al. (83), a preliminary codebook was generated based on common, overarching holistic themes emerging from an initial subset of clinician interviews. Initially, two Black female members of the research team (one undergraduate and one master's level student) independently reviewed two separate subsets of clinician interview transcripts and compiled notes. Both possessed minimal experience and exposure to PCIT based on conducting the clinician and assessment research interviews regarding PCIT. The first author, an Afro-Latina PCIT-IA within agency trainer (WATer), further reviewed both students' notes. The notes were cross-referenced using a similar independent review of a third subset of clinician interviews. The emerging, common, overarching themes, sub-themes, and codes were subsequently used to create the initial codebook.

Once the initial codebook was developed, a third Black, female doctoral-level graduate student trained in PCIT and close to receiving certification as a PCIT-IA Therapist was added to the team. Thus, the final group was a 4-person coding team consisting of one undergraduate, 2 graduates, and a supervising Ph.D.-level WATer trainer, and first author. Accordingly, all coders could pull on their rich lived cultural experiences as members of Black communities in highlighting cultural themes relevant to Black families, and their providers of color. The first author conducted regular coding meetings with the three student coders. Initial meetings were allocated to training on the use of the preliminarily developed codebook. Subsequent meetings entailed coding reviews, reliability checks, and refinement of the codebook in which either new codes were generated, or pre-existing codes were further refined, and or elaborated upon. This iterative process of progressive review of coded clinician interviews, scaffolded into a more elaborate and richer final product that authentically reflected the voices of its participants. The final codebook consisted not only of large overarching themes (22), but further sub-themes (114), and a vast array of unique codes (417) nested within. The codebook was not finalized until all clinician interviews had been coded. Once the final codebook was derived, all previously coded clinician interviews were subsequently re-coded to ensure thorough accuracy, and that all new codes or refined codes were appropriately captured. While the original codebook was created using a bottom-up qualitative data-driven process, codebook refinement utilized a top-down process informed by the first author's wealth of expertise with the PCIT treatment modality, servicing Black families in treatment for over 20 years, and the research literature associated with PCIT and Black families. Thus, this top-down process was consistent with a deductive, reflexive analytic approach for interpreting responses in the refinement of the codebook [(84); see Supplementary Appendix A for a copy of the final codebook (85)].

The initial data analytic plan was to use a codebook approach of thematic analysis for achieving interrater reliability (IRR) of 80% or better agreement. Achieving an IRR of 80% or better would subsequently allow for splitting the interviews to be coded among team members for independent coding. However, because of the complexity and richness of the qualitative data (subsequently lending itself to an equally rich and expansive codebook), achieving a high IRR was difficult to consistently achieve. After multiple rounds of coding interviews and codebook review, and group discussions for reconciling disparities, IRR agreement ranged from 24%–77% with the average being 50% IRR agreement. Therefore, a shift was made to a consensus coding approach, wherein codes were reviewed and approved by at least 3 coders. As previously described, if segments of an interview did not fit into pre-existing codes, new codes were discussed and approved by the coding team, then incorporated into the analysis as coders read and reread each interview. Given the extensive detail of the codebook, a three-person consensus approach ensured a thorough and inclusive coding strategy, where if one coder overlooked the applicability of a code, another on the team was able to capture the essence of the quote.

After coding was finalized, count scores were calculated for each unique code based on the number of clinicians who had a quote that referenced the code. Consistent with the density and richness of the qualitative data from the clinician interviews, and the detailed nature of the codebook, when doing a consensus approach, quotes often contained multiple concepts concurrently. In such instances, those quotes were awarded all applicable unique codes. Therefore, count scores for unique codes were not mutually exclusive of one another. Further, totals were calculated within each interview for the number of times a clinician made a quote applicable to a specific code.

Although there were 22 overarching themes defined in the final codebook, the focus of the current paper was mostly associated with findings regarding the codes in the section entitled “Clinical Training*.*” The Clinical Training theme was analyzed based on those associated codes receiving the highest count scores within each racial group category of clinicians. That is, for each racial group of clinicians, codes that appeared most frequently across transcripts were considered as part of the most prominent sub-themes for subsequent analysis and reporting. Within each racial grouping, count scores for each of the unique codes were classified by their percent rate of endorsement across clinicians as either being in the high tier (>70%), moderate tier (40%–60%), or low tier (≤29%). Quotes that especially captured the essence of the subthemes and codes were subsequently identified for inclusion in the narrative and compared across racial groups revealing both shared and divergent sub-themes. Quotes reported throughout this paper were edited for clarity and readability, while retaining the respondents' original meanings.

## 3 Results

After the completion of coding, researchers had identified 41 unique codes related to Clinical Training (See Codebook in Supplementary Appendix A). These codes emerged due to the nature of the interview questions posed to clinicians, some of which prompted them to discuss their PCIT training experiences within the context of cultural sensitivity and preparedness for working with Black families in treatment. Across the broad thematic domain of Clinical Training there were converging sub-themes among all the racial groups, as well as points of divergence between the racial groups as outlined below.

### 3.1 Convergent sub-themes across clinician racial groups

Two major convergent sub-themes emerged with high endorsement ranging from 80%–100% across all racial groups of clinicians: (1) emphatic affirmation of the good core skill-building training clinicians stated they received in PCIT, and (2) the need for greater attention towards addressing cultural competencies specific to PCIT during training. In addition, although to varying degrees of endorsement ranging from 25%–100%, Black, Asian, and White clinicians shared the sentiment that the climate of the training environment presented as Eurocentric.

#### 3.1.1 Core PCIT training was good

Clinicians across the board spoke laudably about their core PCIT training. When asked to rate their training experiences on a scale from 1–10, with higher scores representative of more favorable endorsements, most participants rated their core PCIT training experience as being an 8 or better. Among White, Asian, and Multi-racial clinicians, 100% rated their core training experiences as being an 8 or better, with 70% of Black clinicians saying the same. The commentary in Table 3 illuminates several aspects of clinicians' positive core PCIT training experiences. They included: (1) the location of training in which clinicians often mentioned graduate programs, internships, and postdoctoral programs, (2) access to diverse populations to train with, and (3) strong knowledge of the manualized treatment protocol.

**Table 3:** Cross cultural quotes regarding the good core PCIT training received.

Black	White	Asian	Multi-racial
70% (*n* = 8)	100% (*n* = 8)	100% (*n* = 2)	100% (*n* = 2)
“I was in a PCIT lab for grad school … [in an area] highly populated with Black people from like different cultural backgrounds. So, I think that's been helpful in just seeing the diversity of Black people, … how they engage in therapy.”	“My training … as a graduate student was great … I really enjoyed … my graduate training or my internship training. [It] was outstanding … That training experience … really shaped me as a PCIT clinician. I still use some of the resources, and practice … training strategies … I learned as a within agency trainer.”	“My training was thorough … I'm so grateful for the training that I received in PCIT and I think it has really served me well in my career … I'm able, to better serve communities, both Black communities, other communities of color, White communities.”	“I'd probably give it like an 8. I got very good training … and kind of know the protocol forward & backwards.”

#### 3.1.2 PCIT trainings lacked attention to cultural issues

While there was a general consensus regarding the high quality of core PCIT training, clinicians reported commonly feeling that the trainings lacked sufficient attention to culture and PCIT's tailored application to working with Black families (See Table 4). Such sentiments were endorsed by 100% of Asian and Multi-racial clinicians, 90% of Black clinicians, and 50% of White clinicians. Some clinicians discussed that issues of culture were not addressed at all, and felt that PCIT trainings should include some cultural competency training specific to PCIT. Accordingly, there was a feeling that the emphasis on treatment fidelity overshadowed taking time to address areas where the model did not seem to be a good fit for the cultural populations that clinicians served at their agencies. Relatedly, others marveled at the limited availability of Black families to work with in their training.

However, while all clinicians at some level acknowledged the lack of attention to cultural competency within the PCIT trainings, Black clinicians in particular noted that they were not expecting their White trainers to have access to such expertise as noted in the following quote:

Yes, so when I was trained I was in a cohort of a lot of people, but I felt like I was the only Black person, and the trainers that were … [there] were not Black either, and so I felt like there was a component missing as far as like culture … nothing … as far as like: ‘this is how you would approach  …  a Black family’. But they didn't have that experience, and they didn't have that knowledge so [you] can't hold that against them. But at the same time, I feel like learning that part would have been most helpful of how me, being Black, … being a woman, … providing PCIT, that was predominantly for White kids [was important for working with] … my families. What does that look like, you know, and things like that? So that was messy, and maybe a component that somebody could do on the side, or something like that, as far as like a training and things like that would be helpful.

#### 3.1.3 Training climate was “White”

Among Black, White, & Asian Clinicians, there was a shared sentiment that within PCIT learning communities, such as conferences or trainings, there was a cultivated climate of “Whiteness.” For clinicians of color, this climate illuminated their “otherness” and negatively impacted their training experiences (see Table 5).

**Table 5:** Clinician quotes regarding the “White” climate of PCIT training spaces.

Black (*n* = 10)	White (*n* = 8)	Asian (*n* = 2)
30% (*n* = 3)	25% (*n* = 2)	100% (*n* = 2)
“When I was in grad school when I went to like PCIT conferences … literally my first PCIT Conference was in Florida, in wherever PCIT was … made, the University of Florida, I think there was like literally two Black people at the whole conference. Like literally. Then my next conference was in Boston. Maybe there's like four Black people … so like when I'm in big PCIT spaces, which I am often, there's no Black people there, & I think for me as a trainee … sometimes that … has been a thing.”	“and all of my trainers were White … ”	“I felt comfortable and confident as an Asian American trainee, but I did notice that my trainers were all White, and the room was predominantly White trainees, so, of course, that shaped the energy, communication style, & overall space during training.”
	“ … Overwhelmingly the people, the therapists involved in PCIT are White women, & … that is a signal to lots of Black families that … this is White people stuff, or I as a clinician, [we] can't relate to their experiences, which is completely valid.”	

Such viewpoints were also communicated through Black and White clinicians' desires for a more visibly diverse representation of PCIT clinicians, trainers, and decision makers. Both Black and White clinicians identified the need for increasing the number of trained providers of color (Black Clinicians = 50%; White Clinicians = 63%), and those who hold higher level positions in the PCIT organization, such as trainers and decision makers (Black Clinicians = 40%; White Clinicians = 25%; See Table 6). The call for an increase in Black PCIT clinicians, emerged at a slightly higher rate for White clinicians, compared to Black clinicians. However, the reverse was true regarding the call for greater Black representation among trainers and the PCIT community leadership. Instead, this sub-theme emerged at almost double the rate for Black clinicians, as compared to their White counterparts (see Table 6).

**Table 6:** Black and White clinician quotes calling for more trained providers and trainers of color.

Need for more Trained Clinicians of Color	
Black (*n* = 10)	White (*n* = 8)
50% (*n* = 5)	63% (*n* = 5)
“We already have … stigma when it comes to mental health services … in the Black community … so, seeing people who look like us is most helpful. The client that's multi-racial, she was like, ‘*I don't mean to be rude, but* … *’* she said, ‘*Are you Black?’* And I said, ‘*Yes,*’ and she was like, ‘*Okay, I just wanted to make sure* … *because I wanted a Black therapist* … *, because* … *‘I didn't want to have to explain why I respond or do the things that I do to someone who doesn't look like (me)*.’ … So that was important for her to have a Black therapist, and I think that is one of the biggest barriers … in mental health in general, [there is] less representation and things like that. So, I think that … would be helpful if we have … African American clinicians and things like that. That would be most helpful for people to know that we're here, and … we're here to help.”	“I think that there [is a] need [for] … more Black clinicians. I think there needs to be a push to recruit more Black clinicians … I think that if more effort can be put into training Black clinicians, then I think that there would be an overall improvement in attrition rates.”
	“I think that we have … got to train more therapists of color, so that if they [clients] would like to have a therapist of color, … that option might be available to them, … and it would be really helpful if we could ask, ‘*Do you have a preference with regard to the race of the therapist that we choose to work with you?’* Wouldn't that be amazing?”
Need for More POC Trainers/and Among PCIT Leadership Decision Makers	
Black (*n* = 10)	White (*n* = 8)
40% (*n* = 4)	25% (*n* = 2)
“I think like based on who you're train[ed] by … , if they understand, like nuances of culture and language, and they have that experience, and they're going to [be] training you in that way … It would have been easier if there were like more seasoned PCIT clinicians who … are Black, and who work with Black families that I can consult with, that I can kind of reach out to. I think that would be helpful.”	“I think just … having that [wording in the manual] be something that … someone looks at. And ideally … not White writers … That are looking at the manual to be like, *how would this translate well to people in our community?* … *What are things that are relatable? Like how can we adjust this language to be more inclusive to sound like what our clients* … *sound like*? … *How they talk at home?* … [that] would probably be my number one thing [to address].”
	“I wonder if, … having more … Black therapists right on the team that develops, or revamps … the details [of the manual] … I think that would be really helpful, and I think that it would make … at least the language piece, because, PCIT is all about language, right? It would make it a little bit more relevant for these families, so maybe they could feel a little more connected, and like … this could be for them right? As opposed to like. ‘*Oh, this is just for some people*’ … even though it's not.”

#### 3.1 4 Similarities across Black and White clinicians for lower endorsed training items

There were four areas that received lower rates of endorsement by Black and White clinicians during their training. First, they reporting having some skepticism regarding whether PCIT would work (Black = 30%; White = 25%). The nature of the quotes included needing additional time to do their own processing to figure out if PCIT would be a good fit for themselves as clinicians. Second, Black and White clinicians reported raising issues of culture during the training (Black = 20%; White = 25%) in the context of the PCIT model. Third, a few Black and White clinicians reported feeling that the training was too time consuming (Black = 20%; White = 13%). Trainees reported conflicts with their regular work responsibilities, and struggling with taking time off to train. Last, while there were significantly favorable appraisals of the overall core PCIT training (as referenced above), a few Black and White clinicians also made reference to a favorable appraisal of their specific PCIT trainers being good (Black = 20%; White = 25%); See Table 7).

**Table 7:** Similarities across Black and White clinicians for lower endorsed training items.

Black (*n* = 10)	White (*n* = 8)
**Cognitive Dissonance during Training**: Trainee needed to do their own process of figuring out PCIT and whether it would be a good fit for themselves as Clinicians or holding a level of skepticism about whether PCIT will work	
30% (*n* = 3)	25% (*n* = 2)
“As … a Black trainee … learning PCIT, and how that was for me, I think that learning it and understanding it, was definitely very important for me so I could also have my own buy-in of … how is this supposed to work? I feel like I'm just kind of giving small nuggets to families, and this is not what they want. I had to know and understand and learn the process … [of] why and what it's rooted in.”	“In terms of cultural sensitivity, I had some questions about how people of color would receive, interpret, and feel about the PCIT model … given by a White Clinician. Related to culture, but not necessarily being White, I also had doubts and insecurities coaching parents since I don't have children of my own, and how they might feel about me not having lived experience raising children.”
Trainee Raises Issues of Culture During the Training	
20% (*n* = 2)	25% (*n* = 2)
“A question I asked when I was getting trained in PCIT was, if it was culturally competent to Black families, because the trainer that I had— I think, was from maybe like the Midwest, or something like a completely different background and location.”	"Race and culture were not part of my training as a PCIT trainee and all of my trainers were White. While this did not make me question the model as a whole, it is something I discussed with my in-agency trainer."
PCIT Trainer was Good	
20% (*n* = 2)	25% (*n* = 2)
"Yeah, I actually really love it [PCIT]. I had a good [training] experience. I … trained with[Trainer's Name], … [who was] extremely culturally aware … I could feel that, like in the training. And so it just helped overall."	"The person that I had who was our global trainer when I was still being certified. I really loved both of the trainers I had."
Time Consuming Training & Conflicts with Regular Work Responsibility	
20% (*n* = 2)	13% (*n* = 1)
“I thought the training,, was a lot. It was 40-hours a whole week. It was a lot, but I like the model. I think it's a good model, but they need to definitely make some provisions for people of color.”	“So I would say my job was supportive on paper, but like in terms of actually … carving out the time for me to do that [the training], they didn't. I still ha[d] the same expectations and it was just … added work for me. So, I'm glad I really enjoyed it, because I wouldn't have stuck with it because it was just like one more huge thing on my plate. So … I think maybe carving out more time for people to properly get trained in it. Maybe that means like offloading some of their case load, or something like that to help trainers actually get through it, because it is demanding,. I think that's a general problem, especially with like social work, case management, things like that … seeing as many people as possible, and kind of the turn and burn mentality, and so I didn't enjoy that aspect of it [the training].”

**Table 8:** Lower endorsed PCIT trainer domain items: Black > White clinicians (3:1 ratio).

Black (*n* = 10)	White (*n* = 8)
Trainer was rigid about PCIT fidelity and Strictly Following the Treatment Protocol	
30% (*n* = 3)	13% (*n* = 1)
“I was trained … by some very strict trainers, so it took us some time to get through, you know, training and certification and stuff, because there were certain things they had to … really beat … [in]to our heads.”	“ … when I was training students … model fidelity was really important to get them certified, and so I was much more likely to just stay on the guidelines and do things in order.”
Trainer Talked about and was Receptive to being Flexible/Adaptive for Culture & Openness/Flexibility of the Trainer	
30% (*n* = 3)	13% (*n* = 1)
“ … my trainer was open to ideas and suggestions. She was also like helping to set up a manual in another language. And so, a lot of our questions were about like, how does this translate to that other language?”	“And so I wasn't sure how much wiggle room there was, I think, the second global trainer I had was like, yeah, you know, you can kind of you know, use different language that would be appropriate for the family.”
“…After Floyd was killed, then … [THE TRAINER] got on a call and said … we want to hear what you have to say, and PCIT is interested in this and that. And she was actually tearful, saying, ‘I'm so sorry that I wasn't listening to you guys. So, I think that after that, that's when they were able to make some adjustments.”	
"Yeah, our whole team the first week we were in training, for … the entire week we were saying, ‘how is this relevant to Black people … How many Black families have used this?’ We questioned it … I'm sure it made the trainer feel uncomfortable, because I don't think that they had a lot of Black families they had used it [PCIT] with, but you know, … they were honest, and said that the recidivism rate was not that great, but you know we think it can work.	

**Table 9:** Black and White clinicians speak about learning when they can be more flexible.

Black Clinicians in Training			
1	2	3	4
“So I noticed a lot of those things [referring to DPICS disparities in coding AAVE] early on as I was getting trained, and … at the time I didn't know that I could be more flexible with that stuff.”	"But at that time they weren't being more flexible, so [in] the training we were … mandated from our job to have to do the PCIT, people didn't really want to do."	“ … if I was recording and say the supervisor was looking at it, they would say, Well, that's a command. And so we had differences in that way. So, after we had the conversation, then they said, ‘You know … there can be flexibility with it.”	“[the trainer's] whole thing was like ‘Black families are going to take longer because of all these other … reasons.’ And in retrospect … I think I struggled with that because it was like, “okay, Yes, all these reasons exist. But does that mean they [Black families] won't be successful in PCIT? Are we not … accommodating or tailoring enough?” … Then midway through my training, another person came to our clinic, and she was like, “Yes, we know. PCIT is hard. Yes, we know it looks like this, but do we do Black families a disservice when we don't hold them to the same expectations that we do other families because we think that all these other things are getting in the way? … that's racist … saying … .because of all these things we're gonna just have you be in PCIT for 35 sessions' where if this was like a White family with 35 sessions, we'd be like ‘no, like what's happening?”
White Clinicians in Training			
1	2	3	4
“I think … the whole point of PCIT is like there's a code for everything, every phrase. And so, I respect that and understand where that's coming from. And I think just again, if there were a way to give a little bit of like clinician discretion for certain things like the intention of a phrase or something like that.”	“I feel like my confidence as a clinician recognizing these barriers is improving, but I also know that I'm someone who really tries to … be a scientist-practitioner, and recognizing what the literature tells us, what science tells us … unfortunately we don't have the most up to [date and] as comprehensive … thorough … science for engagement strategies."	“I didn't know like, how far to stray from the manual, because the manual is like supposed to be followed. So … I don't know whether I … found that sort of sweet spot.”	“I am a big fan of model fidelity, and I feel like it's important. But I also feel like it's important for clinicians and training to know that the clinical judgment is a huge piece of this. I think that you know sometimes that gets lost and just kind of giving clinicians the permission to go off script when needed, is really important … I think that what I've experienced is that trainees who are maybe … too afraid to go off or deviate from the protocol, have less successful results.

Similarly, in matters related to *PCIT Trainers*, there were other lower mutually endorsed trainer items that emerged for both Black and White clinicians (See Table 8). However, Black clinicians tended to have higher rates of endorsement compared to their White counterparts across the *Trainer domain areas* in a 3 (30%/*n* = 3) to 1 (13%/*n* = 1) ratio. Those *Trainer domain areas* included perceptions of the *trainer's rigidity* in strictly following the protocol, while other clinicians cited experiences of their *trainer's openness to discussing cultural issues* in PCIT, and demonstrating a *willingness to explore cultural adaptations*.

Consistent with the discussed themes around openness and flexibility, clinicians discussed that when they were less experienced, there was uncertainty regarding how much latitude they had to exercise flexibility with the PCIT model for retaining their families, and when to exercise that flexibility. (Black = 40%; White = 50%; See Table 9).

However, with time and experience clinicians developed a feel for where and when they could exercise some flexibility in their clinical discretion, with 70% of Black clinicians reporting that they *exercised flexibility in PCIT*. The same was true of White clinicians wherein 87.6% reported utilizing a more flexible approach in tailoring PCIT to Black families following training.

### 3.2 Divergent PCIT clinical training themes from Black and White clinicians

There were also points of divergence among clinicians. However, such qualitative analysis was limited to Black and White clinicians with middle to lower tier percentage endorsements. Black clinicians expressed concerns around (1) *Power Imbalances* between themselves and their trainers (40%), (2) *Stylistic Communication Differences* between themselves and their White trainers (20%), and (3) listed a variety of *disruptive barriers to PCIT training* (cumulatively 40%) including (1) challenges of managing trainings during COVID-19 disruptions (10%), (2) desires for additional supervision (10%), prohibitive costs of PCIT training (10%), and low caseloads (10%).

White clinicians on the other hand, were more focused on first, *increasing their cultural competency* in working with Black families (50%), with one clinician endorsing their feelings of inadequacy with respect to being able to coach following the training. Second, White clinicians also reported greater instances of cultural incompetence in which they reported *not raising issues of culture in treatment* (60%), as compared to Black clinicians (10%). Third, White Clinicians also seemed to possess a sensitivity to their privilege in *having access to PCIT training* (25%).

#### 3.2.1 Black clinicians divergent sub-themes

With respect to training experiences, 40% of Black Clinicians reported feeling a *power differential* between themselves and their training supervisors. Underlying such conflicts were several key factors, in which Black Clinicians felt: (1) more culturally competent and knowledgeable than their White PCIT trainers regarding how to clinically proceed with their Black clientele, (2) that the PCIT training climate was not welcoming of the valuable cultural wisdom they brought to the table, that was grounded in their insider perspective and experiences as members of the Black community, and (3) there was an added layer of fear regarding the caustic consequences of going against a system that had power over their career aspirations of becoming psychologists.

Such differences in power were further compounded when being trained in an academic setting, in which the PCIT trainer also served in a dual relationship as their academic thesis/dissertation advisor. Given these three areas of concern, 20% of Black clinicians admitted to withholding their voices over concerns about advice given by their White trainers on how to clinically proceed with their Black families who were engaged in therapy. Some Black clinicians discussed opting for silence, with the aim of offsetting any anticipated negative consequences for potentially voicing disagreement with either their trainer, or challenging of the PCIT model. Additionally, such silence was chosen even in light of the potential for those challenges to create opportunities for better culturally informed PCIT care, given the Black clinicians' wealth of knowledge about Black culture, grounded in their lived experiences.

For other Black clinicians who choose to speak up, they reported sometimes feeling not heard by their trainers, or that their opinions regarding culture were not being respected. In Table 10 two separate Black Clinician's describing their sensitivity to the unsafe supervisory climate, where they did not feel free to exercise their own voices in imparting their cultural knowledge regarding Black communities, and its contextual influence on the PCIT treatment. While another Black clinician recounted their attempt to assert themselves with their White trainer, by standing their ground to protect and preserve the therapeutic relationship with their Black client.

**Table 10:** Black clinicians' perceived power differential between themselves & training supervisors.

Black Clinicians		
1	2	3
“It wasn't always room to be like, ‘Well for the Black family that I have, this is what's different,’ or ‘This is how we do this,’ you know? And as a trainee … in a White system with White people holding the power to whether or not I would get my dream of becoming a Black psychologist … it didn't always feel like a place to be able to say ‘this doesn't seem right for a Black family.’”	“That's a double-edged sword, though. My trainer was also my thesis … dissertation chair, and was in charge of my career. You know? … But she was sweet.”	"I did not have one single parent that was willing to allow me to record. And my supervisor was getting on me, saying, oh … you know I'm gonna have to sit in. We're gonna have to record.,, I'm saying … ‘My parent is super shy. I'm telling you … she is not going to want you back in that other room observing her. And you're going to ruin my relationship with her, because I told her that nobody is watching it … No, you're not going to do that.’ So that caused some friction between [me and] my supervisor. I mean it wasn't nothing major. She got it … When we got on a call she said, ‘Well, we're gonna have to find another way to reach fidelity.’"

Relatedly, 20% of Black clinicians noticed *stylistic differences in communication* and social interactions compared to their trainers, which served as potential communication barriers, that further distanced them from the PCIT training and community as illuminated in the following quotes.

“So I think that after that [the death of George Floyd], that's when they were able to make some adjustments and recognize that we speak differently. We interact differently … .so you need to be aware that what we say, has relevance. We know our people better than you do. So, if we say that that's not a ‘command,’ then that's not a ‘command,’ you know.” (Clinician 1)

“…especially with the language and what we're considering ‘praise’ vs. ‘neutral talk’” (Clinician 2)

Consistent with Black Clinicians' sense of cultural competency in working with Black families, one clinician reported leveraging their cultural competency to build a strong level of trust with their Black clientele that enabled them to become certified before several others in their White training cohort.

“One thing I noticed when … our whole team [did the training]. It's like 6 of us … I picked people [cases] that I knew, where everybody else just picked new cases they were assigned, and they were really struggling … I had successfully completed one and then, when I came back after 4 months [of medical leave], I successfully completed the second one before any of them had completed any of their cases … I think It'll help them to see that you really need to have a strong relationship with those African American families before you start … PCIT. They have got to trust you. They've got to know that you know that the things you've taught them already has worked, and so they trust you to move to this next step, because if you just take them straight in from a brand-new case, I don't think it's going to be that successful.”

Forty percent of Black clinicians also remarked about instances when their training, although good, was less than ideal due to a myriad of barriers including: (1) prohibitive costs of training (10%), (2) challenges in securing a PCIT caseload, (3) challenges of managing trainings during COVID-19 disruptions (10%), (4) desires for additional supervision (10%) (See Table 11).

**Table 11:** Barriers to PCIT training for Black clinicians (*n* = 10).

Training is Expensive	Low Caseload	COVID Barriers	Felt like they had less “Hands-on” Supervision during their training
10% (*n* = 1)	10% (*n* = 1)	10% (*n* = 1)	10% (*n* = 1)
“ … I think the process of getting trained is not as accessible … there are not very many Black PCIT therapists. It's expensive … for a lot of us that work for organizations or community mental health. We're not given time to be able to do a training like that. I mean, you have to take off a whole week and … that's billable hours that you're not going to be able to get [paid for] … so the focus on professional development isn't always as important in systems that are devoted to making money.”	“My dropout [rate] right now is like super low … But I think in the beginning [it] wasn't— I wasn't always clear about that or in the training process, and the different institutions that [I] trained in. I think we just assume[d], because pediatricians will say your child needs PCIT and then you [would] come to it [PCIT].”	“ … the way I was trained. It was during COVID, and so there was a lot of barriers, and a lot of adjusting that I had to go through with the training process, and so [it] kind of stumped a lot of my learning.”	“So, it was a postgraduate fellowship opportunity that brought me here and allowed me to be trained in PCIT … I know for the supervisors that I have … everybody is busy … I think to be able to really have the opportunity to supervise those as best needed, you could call on them. But you know, there were times, and I'm just being honest, that we're kind of just thrown into doing a case on our own, and not having … a true step by step … following up and really observing … me do a whole teach … it's kind of just in pockets … And I think … both supervisors … are done with their Level 1's now, you know, that training for them … I know that [this is] the best that they could [do]. But you know, that just felt like there definitely were times where I was like, I don't know what I'm doing. Is this right? I kind of feel like a fish out of water.”

#### 3.2.2 White clinicians' divergent sub-themes

##### 3.2.2.1 White clinicians' desire for greater cultural competency

On the other hand, White clinicians in greater proportion, reported that they did not bring up issues of culture in treatment just by the simple process of adhering to the PCIT protocol, especially in instances when they were training others. The failure to raise issues of culture in treatment by 62.5% of White clinician was a stark contrast to the only 10% of Black clinicians. Quotes by White clinicians suggested that despite their earnest investment in wanting to demonstrate culturally competent care to their Black families, some White Clinicians felt insecure about their implementation abilities (see Table 12). Accordingly, White Clinicians focused more on acquiring cultural competency and resources that would bolster their ability to implement culturally appropriate PCIT interventions with Black families.

**Table 12:** White clinicians wanting more cultural competency training.

White Clinicians Wanting More Cultural Competency Training			
1	2	3	4
“I had questions about how to best relate the skills in a culturally relevant manner.”	“I would love to know about ways to be able to kind of bring that [culture]up. If I feel comfortable bringing it up a little bit more [that would help], because I kind of don't know where to start.”	“I feel like in the intake. It's like, okay. It's written [in the manual] so like I'm going down a list of questions, but like I would love … to have like a more natural way to kind of talk about that [culture] to let families know, like I'm still here to hear this, and I'm still here to support you, and help as much as I can on my end.”	“I can be open and affirming and validating, but I can't talk to that specific experience as a White person. So [I would like] maybe some sort of handout about like parenting style or multi-generational families, like how to handle it when there's one parent in treatment, but grandma is like, really not on board & trying to do things really differently. Or potentially, like some videos that, in particular, talked about the experience of doing PCIT for Black families.”

White clinicians expressed wanting to be better educated about the intricacies of tailoring PCIT to Black families in ways that are not only culturally appropriate, but are quite nuanced and often differ from their phenomenological perspective as White clinicians, as represented in the following quotes:

“You know, the mom had endorsed concerns for domestic violence and fear for her child, and obviously how we wanted to be protective of that child. And I'll never forget this, you know, we talked about safety planning: what she could do if things escalated to protect herself and her child. And this was prior to George Floyd being murdered, and she said something about ‘I'm not calling the police. Why would I call the police?' And I'm sorry if I get emotional about it. It was just I remember going home that day and feeling like ‘how could someone not call the police?’ It was something that really stood out to me … That's something I've always been considerate of … ’ how do we link families with resources … [that they] might be disproportionately already subjected to some of the horrible discriminant practices we know occur? … You know, I had a sense of White guilt when I would never fear calling the police. But I know that obviously … impacts parenting as well.”

“I had a family that I was working with, who I taught them ‘labeled praise’ and the mom, she found the whole ‘label praise’ thing very powerful … She was out on the playground, and she lived in government housing, and she was using ‘labeled praises’ … And she had a group of 2 or 3 parents from the public who came up to her and said, ‘why, you always bragging on him? Why do you think he's better than everybody else?’ And she came back in, and she was really conflicted. She said these skills are making her look bad in her community, and that she was considered to be boastful because she was using [PCIT skills]. And so, we stopped any kind of public praising in front of family members or extended family, and we went to whispering private ‘praise,’ but I didn't see it coming. [I] just had no idea.”

Addedly, when first implementing their newfound PCIT skills, some White Clinicians acknowledged using a trial-and-error approach in which they acquired their cultural knowledge via increased clinical experiences in working with families, and noticing the greatest gaps in knowledge were with minoritized families:

“I was trained in PCIT at a time when culture was not emphasized. I had to learn through experience, with a lot of trial-and-error. I learned that each family, regardless of race/ethnicity, enters PCIT with unique values and parenting backgrounds. But, I clearly had the most challenges relating to U.S. families who were from minoritized backgrounds.”

As such, White clinicians wanted more specific cultural advice about how to tailor PCIT to Black families from their PCIT trainings, and additionally reached out to their Black colleagues thereafter:

“There's only one other person at my agency that is trained in PCIT, and she's Black, and so we have done a lot of joint sessions … Whenever there's a topic that was a little bit difficult, she would kind of step in and know when to, you know, respond to some of those questions. But I consult with her quite a bit about things.”

One Black clinician also echoed how PCIT training needs for White clinicians needed to focus on increasing their cultural competencies in working with Black families.

“Yeah. I think that the White therapist need(s) to get better training on how to work with Black people, and that's not just for PCIT … It's easy just to put out a model and say, ‘try it’, but if you don't know how Black people function and work, and what their priorities are, it's hard for you to provide a service and know what our people like. You know?”

White clinicians also made reference to using content emanating from the PCIT-IA listserv, town halls, conferences, and monthly collaborative calls for continuing education for better understanding of how to address issues of culture in PCIT following their initial PCIT training.

##### 3.2.2.2 White clinicians' awareness of their privilege in accesing PCIT trainings

Another racial difference was that contrary to their Black counterparts who voiced financial hardship with receiving training, 25% of White Clinicians acknowledged their advantage in being able to secure the financial support from their employers in receiving PCIT training. One clinician noted, “*I was trained courtesy of the city* … *in an initiative, which was designed to improve capacity within the city.”* Another clinician gave her last job credit for paying for her training in PCIT, *“and you know, making [PCIT training] happen* … *,”* when she was *“overworked, over-tired, & underpaid.”* Additionally, there was one White clinician who focused on the need for greater resources in marketing and funding to be allocated especially for training clinicians who serve underserved Black communities.

“…I think maybe marketing it more to clinicians that serve communities that are primarily … more Black residents. I know there are clinicians in [LOCATION DEIDENTIFIED], because one of them train[ed] me. But there should be more, and I guess, doing whatever they can with either grants or funding to like market to experienced clinicians who represent the communities that they serve, like actually can serve in those communities, I think [it] would be best. I personally work in [LOCATION DEIDENTIFIED], I don't know of a lot of clinicians that are certified, but I think like trying to do whatever can be done to like train more clinicians that serve Black families would probably be a start.”

### 3.3 Black and White clinician provider cultural competency

Given the focus on cultural competency for these clinicians, trained in PCIT, it seemed important to have an objective appraisal of their overall cultural competencies as measured by the 20-item self-report measure of provider cultural competence [PCC; Cultural Competence Instrument for Primary Care Providers; (68)]. A series of independent two-tail t-tests were conducted to examine racial differences among Black and White clinicians across all five domains of provider cultural competency (PCC) for Black and White clinicians. Asian and Multiracial clinicians were not included in these analyses as their sample sizes (*n* = 2 for each group) were too small in each racial category. With respect to PCC, there was a trend of marginal significance for Black providers expressing greater overall cross-cultural competency [*t*(16) = 1.9, *p* = 0.07, d = .90; Black *M* *=* *4.97*, White *M* *=* *4.69*] with a large effect size (d = .90). More specifically, Black clinicians expressed a significantly greater concept of one's cultural identity [Concept of Culture Subscale; See (68)] [*t*(16) = 3.18, *p* = 0.005, d = 1.51, Black *M* *=* *6.00*, White *M* *=* *4.25*] and ability to implement cross cultural care [Cultural Care Subscale; See (68)] with their clients [*t*(16) = 3.2372, *p* = 0.005, d = 1.54, *Black M* *=* *6.00*, White *M* *=* *4.25*]. Both findings for concept of one's cultural identity (d = 1.51), and cultural care yielded significantly large effect sizes (d = 1.54).

However, in all other domains of provider cultural competency (PCC), there were no racial differences found. Both Black and White clinicians acknowledged the *inequitable healthcare disparities* that exist for marginalized clients [*t*(16)= 0.79198, *p* = 0.44 (NS); Black *M* *=* *5.8500*, White *M* *=* *5.6875*], endorsed using a *patient centered communication approach* to care [*t*(16) = −0.79057, *p* = 0.4408 (NS); *Black M* *=* *4.1250*, White *M* *=* *4.3125*], acknowledged awareness and the important role that *diverse beliefs in cultural folklore* play in clients' personal healing practices [*t*(16) = −0.02132, *p* = 0.9833 (NS); Black *M* *=* *4.620*, White *M* *=* *4.625*], and felt the *client's cultural context was relevant* for understanding their presenting complaints [*t*(16) = −0.79057, *p* = 0.4408 (NS); Black *M* *=* *4.1250*, White *M* *=* *4.3125*]. Therefore, consistent with the thematic racial differences for White clinicians, there is a sense of knowing the key core elements of cultural sensitivity and compassion, and wanting to be culturally competent, but being uncertain how to convey and implement those competencies in culturally sensitive ways with their Black families. Black clinicians did not report this same uncertainty. In fact, Coates and colleagues (15) reported that these same Black clinicians noted feeling culturally knowledgeable and proactively taking steps to culturally tailor PCIT in their treatment with Black families.

## 4 Discussion

### 4.1 Strength of training in PCIT's core principles

Consistent with the robust findings regarding the effectiveness of PCIT (5), all PCIT clinicians interviewed endorsed positive perceptions of the core PCIT training. Clinicians emphasized the strong fidelity to treatment in their training that enabled them to master the protocol and skills, whereby they left training with long-lasting competencies, and were similar sentiments echoed by other PCIT training implementation studies (55, 73, 86, 87). It is noteworthy that the quotes offered in the current study tended to be more general in nature, offering minimal reference to the specific aspects of training that were most beneficial beyond rigor. However, in other training implementation studies satisfaction seemed most associated with the supervisory and consultation process for those who engaged in those aspects of their training (73, 87) where specific reference was made to their learning from other clinician's cases, the expertise of their trainer, and the constructive feedback offered in very positive and encouraging ways (30, 73). Thus, PCIT-IA should applaud their stellar accomplishments in solidifying the core rudiments of PCIT training, particularly with the more commonly used *cascading model* approach that entails a 40-hour preliminary training, followed by a 2-day advance training several months later, and a yearlong biweekly consultation, where the greatest amount of skill learning was noted to take place (55).

### 4.2 Perceptions of PCIT trainings eurocentrism & need for cultural sensitivity

However, in an increasingly diverse cultural landscape, whereby clinicians are being called upon to serve a diverse clientele, clinicians expressed concerns regarding how PCIT is perceived in terms of its Eurocentric origins and nature. That is, as a “White” treatment made for “White” clientele as more generally noted by clinicians. [see Table 13 regarding PCIT more generally (15)].

**Table 13:** Black and White clinicians' quotes regarding the Eurocentric nature of PCIT.

Black Clinicians (*n* *=* *3*)^a^		White Clinicians (*n* *=* *2*)^a^	
1	“I don't love PCIT as a Black clinician. I don't love it. It feels again. There's a qualitative piece to it … PCIT honestly feels very White.”	1	“…I'll say talking about … use of PDI. I … had a mother who … . very enthusiastically stated the idea that … this is … not going to work in my home. Or this … is like a White parenting perspective … ”
2	“… I just try to be honest about the origins of PCIT, right, about the fact that it was founded by … White women in a primarily … White rural homogenous area, and that the language came out of that"	2	“But I think in the very early days of PCIT, that the evidence-base was from mostly upper middle class, white families, and so I have always been mindful of that in my work with non-White families, particularly Black families.”
3	"But sometimes what we are encouraging, is what PCIT goes against [in] the culture, if that makes sense?”		

a All quotes offered were by independent individual clinicians for a total *n* = 5 Black and White Clinicians.

In a parallel process, the current qualitative findings speak to Black clinicians' mirroring similar concerns regarding their training spaces being predominantly Eurocentric (see Table 5) and some not feeling a sense of belonging to confidently and freely exercise their voices within the PCIT community, which may be viewed as “*White Spaces*” they must strategically navigate as a marginalized group (88–90).

Subsequently, some Black and White Clinicians expressed ambivalence over PCIT's continued use with Black families beyond their initial training (See Cognitive Dissonance in Table 7). Although clinicians could see some value in the treatment, Clinicians questioned PCIT's ability to meet the unique cultural needs of Black families, given its perceived “Whiteness” (15), as well as questioned its stylistic fit for themselves as clinicians. Consistent with Festinger's (91) theory of cognitive dissonance*,* clinicians experienced a level of discomfort with the incongruence of seeing PCIT's value, with their own uncertainty regarding whether the model would be suitable for Black families, and/or a good fit for themselves as clinicians. Subsequently causing some clinicians to seek out ways to become more comfortable with the model in order to comfortably implement PCIT within a cultural context for Black families.

Such reported trepidation by clinicians during their training, signaled the need for trainings to be more culturally sensitive (See Table 4) to ensure greater buy-in across racially diverse clinicians. Consistent with other reports calling for PCIT to attend to cultural differences (15, 4), clinicians pointedly stated that PCIT trainings lacked attention to culture. Without greater attention to cultural sensitivity in the PCIT trainings, Eurocentric perceptions of PCIT, threaten PCIT's ambassadorship to ethnically minoritized communities and their sense of belonging. With the aim of broadening PCIT's significant impact as the seminal standard for treatment for disruptive behaviors among Black families, buy-in from clinicians during their trainings is paramount. Clinicians, especially Black clinicians, must feel that sense of belonging for Black families, if they are to alleviate their cognitive dissonance to readily step into PCIT ambassadorship roles within Black communities, for ensuring greater PCIT dissemination. In fact, in one study when PCIT training was culturally adapted to Black and Latine clinicians serving Black and Latine families of Autistic youth, clinicians experienced their training space as culturally validating when their racially/ethnically matched trainer owned a space that was traditionally viewed as “White.” (30, 92).

**Table 4:** Quotes depicting clinicians' feelings that PCIT trainings lacked attention to cultural issues.

Black	White	Asian	Multi-racial
90% (*n* = 9)	50% (*n* = 8)	100% (*n* = 2)	100% (*n* = 2)
“I'm just like, you know, this doesn't account for the cultural norms. You know my agency, a lot of my families are Black, like I gotta be able to make adjustments.”	“I was trained in PCIT at a time when culture was not emphasized. I had to learn through experience, with a lot of trial & error.”	“Now I'm like reflecting on my training, and really not that much cultural adaptation– adaptiveness was done in my training … not much cultural considerations were applied to the training.”	“My whole training year … which is kind of like the quintessential kind of training site, we had no Black families for PCIT, which was definitely something I was pretty shocked about.”
	“I feel like we should probably incorporate a lot more training opportunities for PCIT clinicians to kind of help be more culturally competent, specific to PCIT. I think that would be really helpful”	“When I was working with the Black families that I had, there were certain things that came up that … didn't … come up earlier in my training,”

“It was a nice space to be able to go … with … White clinicians present and … respectful and allowing space. That was a refreshing experience, because, sometimes it's just not the case. Going back to that cultural humility piece where I wasn't having to deal with accommodating White discomfort, the White clinicians that were there made room for things and didn't make it about themselves, which is refreshing because they allowed [THE BLACK CLINICIAN TRAINER] to lead. Even though they were in a position of authority, they were able to just be present and attend like everybody else.”

### 4.3 A need for greater Black representation in PCIT

PCIT clinicians spoke to the need for PCIT as a whole, and specifically in its training spaces, to become more visibly culturally diverse in its clinical representation (15), and in the face of its clinicians and governance/leadership (See Table 6). Moore, Coates, et al.'s (64) research found that a majority of Black clientele expressed a preference for working with racially concordant providers. Consistently, Black clinicians in the current study reported experiences of being sought out by Black clients based primarily on their shared racial background. A quote illuminated Black caregivers' desires of not wanting to expend valuable treatment time having to explain the Black lived cultural experience of how discriminatory systems provide context to their presenting treatment concerns. The time saved bypassing such explanations would allow for expedited rapport building; thereby, enabling concordant providers to quickly move directly to problem-solving solutions, as is the treatment preference for many Black clientele (93). Also, above and beyond rapport building in PCIT, there may be cultural translations of PCIT concepts that are germane for cultural buy-in among Black families, which some Black PCIT trained clinicians already report doing with their Black families outside of their PCIT training experiences (15). Accordingly, racially-concordant treatment literature alludes to some positive benefits for Black families, if they were able to partner with Black PCIT providers emanating from their communities, who would bring a wealth of lived experience to bolster the therapy (62, 64).

As previously mentioned, the clogged clinical pipeline has negatively impacted healthcare service provision by Black providers (94–96), and EBTs, like PCIT, are not immune (55). Similar to findings that Black families perceived PCIT as White (14, 15), our study findings indicated that clinicians also described the training climate as “White,” noting the prevalence of White PCIT trainers and trainees. Clinicians across racial groups further called for increased representation of people of color in leadership. To that end, part of being culturally sensitive to the needs of Black families involves having more Black clinicians and trainers who bring their lived experience to the application of PCIT into relevant spaces. Again, taking such an approach not only supports equity, but has implications for broadening PCIT's reach and sustainability. PCIT training that is deemed culturally competent by Black providers, may ameliorate Black family recruitment and retention issues by enhancing the role Black clinicians play as ambassadors of PCIT services within Black communities, and serving as peer mentors to their non-Black clinician counterparts, as is suggested as being done in the current data.

Additionally, while a good portion of both Black and White clinicians expressed a desire for the greater presence of Black PCIT providers, rates of endorsement were slightly higher for White participants at 63% percent, vs. 50% for Black PCIT clinicians. Some speculations regarding this very minimal difference between the two racial groups may lie in racial disparities of privilege. It's possible that White clinicians owning their privilege in the spirit of advocacy and allyship, may have felt more comfortable expressing such comments that might be perceived as more challenging of PCIT norms. Accordingly, White Clinicians' slightly higher endorsement for more Black PCIT providers may have also been reflective of feeling less culturally competent to deliver PCIT to Black families, as noted in the quantitative findings, given the perceived lack of shared cultural worldviews, as respectful outsiders of the Black race. Further corroborated was also found in the qualitative findings with one White clinician noting her deferring to her Black colleague's expertise in co-therapy with Black families. Additionally, research suggests that White individuals are generally reticent to engage in racial discussions, unless such openness is first modeled by Black individuals in their interchanges (97). Such narratives in tandem with a potential sense of responsibility for not wanting to do harm, may have caused more White clinicians to specifically express a greater need for Black PCIT providers, emphasizing their slightly greater focus on frontline service delivery. However, with respect to the call for greater Black representation among trainers and positions of leadership within the PCIT community, it was noteworthy the reverse was true, in which a greater percentage of Black clinicians (40%) endorsed this, over their White counterparts (25%). Such findings indicated that Black clinicians had their eyes not only on frontline service delivery, but solidifying their sense of belonging within the PCIT community as invested stakeholders whose voices might have greater impact on shaping PCITs applications with their communities. Additionally, there was a sense of seeking community within a community in networking with other experienced Black mentors and role models who had successfully integrated PCIT into treatment with Black families.

The racially concordant provider literature also lends support to increasing the number of Black PCIT-trained clinicians to meet the needs of Black families who have preferences for concordant providers (63, 64). Increasing the opportunity for Black racial concordance between providers and clients could potentially positively impact Black families' treatment engagement. Though no research to date has measured treatment duration and outcomes in conjunction with provider-client racial concordance among Black families doing PCIT, there is some suggestion that Black provider concordance may benefit Black families when it does occur. In another paper from the larger dataset, Coates et al. (15) highlighted that Black PCIT clinicians intuitively feel they know how to tailor and translate PCIT concepts, and take the initiative to do so with Black families in PCIT treatment. Increasing the number of Black PCIT clinicians and trainers could, therefore, serve to overcome other obstacles clinicians identified, like those related to providers' lower graduation rates in their work with Black families, which in turn impacts certification.

### 4.4 Promoting cultural competency needs for clinicians in PCIT trainings

Based on the qualitative feedback, the need for cultural attunement, sensitivity and competency development seems to be operating at two intertwining levels. One level speaks to PCIT's culturally sensitive training for clinicians, who will be the frontline workers serving the Black community, and the second speaks to the larger overarching goal of PCIT's cultural suitability for Black families as a relevant treatment modality. Based on these two operating levels, PCIT trainings must utilize a culturally tailored framework that speaks to the unique, inherent cultural needs of Black families: 1) So, clinicians will feel culturally competent and bought-in that PCIT can indeed make a difference with Black families, and 2) as PCIT ambassadors, clinicians will be better able to promote greater receptivity among Black families to remain in treatment long enough to reap the benefits of this gold standard EBT for disruptive behaviors. For PCIT clinicians to meet the needs of Black families, PCIT trainings must address the identified pervasive undercurrent of unmet needs related to building cultural competencies within the PCIT training framework. Especially, as the current data show that clinicians want their trainings to help them feel culturally competent in responsibly and ethically implementing a culturally attuned PCIT intervention.

### 4.5 Racial difference in cultural attunement training needs for Black vs. White clinicians

At the level of clinical training, though there is a consensus regarding the need for culturally competent training within a PCIT framework, the data speak to differing needs for White clinicians vs. their Black counterparts. In true allyship fashion, White clinicians expressed wanting to be better educated about the intricacies of tailoring PCIT to Black families in ways that are not only culturally appropriate, but are quite nuanced, and often differ from their phenomenological perspective as White clinicians. From both the qualitative and quantitative data we see that White clinicians are mindful of cultural competency needs, and endorse its critical enactment in mental health client care. However, White clinicians appear to struggle with translating their good intentions into practice. That is, when first implementing their newfound PCIT skills with Black families, there is trepidation regarding a trial-and-error approach. Such sensitivity conveys a laudable sense of responsibility for the unintended, and sometimes inevitable missteps White clinicians may make, as they themselves are learning the treatment modality, which can have high and costly stakes for the Black families clinicians work with.

This motivation served as a catalyst for seeking additional training supports, like those resources referenced by White clinicians in this sample. While more support was needed, clinicians identified existing supports as coming through a variety of additional continuing education training opportunities made available through PCIT-IA (Conference and workshop presentations, the listserv, and the Town Hall during the BLM social movement). Also as discussed earlier, White clinicians sought out additional support through consultations with Black colleagues in shared workspaces. White clinicians expressed seeking this consultation hoping that their peer mentors of color could guide them through the nuts and bolts of culturally sensitive PCIT implementation with Black families, given their insider perspective as members of the Black community. Endorsement of a belief in their Black colleagues' cultural competence based on racial identity and lived experience, parallels the perspective of Black clients seeking treatment (64), and is supported by data highlighting trends of higher provider cultural competency among providers of color (68). The quantitative findings in the current study also suggest that White clinicians' assessments of their Black colleagues expertise may have been accurate, given that Black clinicians endorsed possessing marginally higher overall provider cultural competence, and were significantly more self-aware of their own cultural identity, as well as confident in knowing how to engage in cultural care.

For Black clinicians going through PCIT training, the narrative foci of their responses were quite different than their White counterparts. A good portion of the Black clinicians interviewed reported experiences that were fraught with concerns over power imbalances with their PCIT trainers/ supervisors, as well as perceptions of a training climate where they felt like outliers. There were concerns about whether traditional systems of oppression and subjugation would be enacted through the training process, preventing them from getting certified. Further, PCIT training in academic settings where PCIT trainers were also academic advisors presented an added layer of concern regarding potential barriers to their programmatic completion, or accomplishment of their professional career goals. Accordingly, there was a perception that there was no place for Black clinicians' voices to be heard or respected. Because of this lack of space, some opted for silence to offset anticipated negative consequences of potentially voicing disagreement with their trainer or challenging the PCIT model. Here, it is important to note that some Black clinicians chose silence despite the potential for those challenges to create opportunities for better culturally informed PCIT care, given their wealth of knowledge about Black culture, grounded in their lived experiences. Even in cases where the Black clinician did speak up and held firm to their convictions in doing what was in the therapeutic interest of their Black client, it was not without consequence: at least one Black clinician expressed sensing the tension between themselves and their trainer.

This proclivity for Black individuals to remain silent in predominantly White spaces has been referred to as identity shifting (98). For Black females, identity shifting is a strategy for promoting professional advancement in predominantly White Spaces, by either speaking or behaving in a fashion that is in alignment with White norms and expectations, while suppressing mannerisms of speech, attitude, or behaviors that would be associated with one's Blackness. Moreover, Dickens & Chavez (90) describe a phenomena they refer to as the “Frozen Effect” in their qualitative study examining identity shifting in early career Black females navigating predominantly White professional environments. In the context of perceived discriminatory experiences, Black females may opt to remain “silent,” become “psychologically paralyzed,” or “mentally checking out in conversations in predominantly White social or professional environments.” By retreating “into isolation and responding with either concise or no replies when communicating with their White colleagues,” Black professional females were able to offset the potential risks to their career advancement (90). As a result, they would adjust the tone and volume of their voice and subdue their presentation to overcompensate and offset negative stereotypes, such as being “angry” ascribed to them, subsequently, negatively affect their professional persona, relationships, and opportunities for professional advancement. Franklin (99) proposed that such professional strategies are enacted when members of a marginalized group feel undervalued or ignored, and coined the term “invisibility syndrome.” Wong et al., (100) mirrored similar findings in a qualitative study of 51 interviews of ethnic minorities navigating predominantly White spaces in an academic setting.

On a more positive note, despite these instances of feeling guarded about using their voices in training, although at a lower threshold of endorsement, there were some instances when Black clinicians reported raising issues regarding culture in training, comparable to their White clinician counterparts. Equally noteworthy, was that nearly a third of Black clinicians described their trainers as being open and exercising flexibility with making adjustment for culture, as compared to only an estimated 10% of White clinicians. Such findings speak to the slow, but positive directions PCIT-IA is taking to become more culturally responsive within trainings. Quotes depicted PCIT trainers' openness to receiving feedback from clinician trainees, self-corrections in cultural missteps, and demonstrated examples of flexibility in accommodations to support Black families.

### 4.6 Training in exercising of clinical flexibility with the protocol in service of Black families

Such slow and steady gains towards flexibility, especially resonated with clinicians who appreciated the nuances of balancing flexibility without challenging PCIT treatment fidelity. This was especially in the juxtaposition of the perceived rigidity of the PCIT training reported among a third of Black clinicians, as compared to an estimated 10% of White clinicians. So, while some clinicians acknowledged that such nuanced skills tend to come with increased experience, clinicians, overall, expressed a desire to receive further culturally tailored training to increase their understanding and skill level for how and when to exercise more nuanced flexibility with Black families, while still maintaining PCIT treatment fidelity. Those calling for more cultural training in exercising PCIT flexibility, was seen as a benefit by all clinicians, spanning a range of experience levels and racial backgrounds. That is, either close to, or equaling 50% of Black and White clinicians reported wanting additional training in understanding when they could exercise flexibility with the PCIT protocol to accommodate their Black families, when they felt some aspect of the therapy needed adjusting. Thus, it seems prudent to examine the possibilities for accelerating this tutelage through the dissemination of culturally tailored PCIT training that benefits all clinicians, regardless of race. Such findings are demonstrative of clinicians' sensitivity to the delicate tension between the inherent cultural needs of Black families, the space in the protocol that was intended for experienced clinicians to exercise their clinical judgement, while also respecting the very clearly expressed need for model fidelity during trainings. However, clinicians felt unclear of how to balance those two very important goals. This is, especially in the context of (1) wanting to help Black families, which is impossible to do if they prematurely drop out of treatment because clinicians are unaware of how to tailor the model to their families' needs, and (2) while many clinicians are still striving to hold on to their PCIT caseloads for ensuring certification requirements.

### 4.7 Addressing racial differences in certification challenges

Thus, a lack of cultural diversity among trainers, as well as the lack of opportunity to work with Black families in particular settings, may obfuscate the certification process, particularly for Black clinicians. In tandem, this study's findings underscore the need to remove the obstacles that limit more clinicians of color from being PCIT trained and certified, such as funding for training access. The data further illuminate the disparity between Black clinicians who cite financial obstacles to training, as compared to their White counterparts, who expressed more privilege in having their training funded by their employers. This disparity in certification completion further highlights the chasm perpetuated by PCIT's Eurocentrism and speak to one of the widely cited etiologies behind the clogged clinical training pipeline for providers of color (96, 101–103). Relatedly, slightly more Black (20%) vs. White (13%) clinicians reported associated concerns regarding the time constraints of PCIT training creating conflicts with their work schedules, and being able to complete their regular demands of their employment, without sufficient support from their employers, which speak to common concerns regarding PCIT implementation efforts (73, 87), and are believed to produce favorable outcomes when partnerships with agency stakeholders are included (55).

## 5 Future directions for an adapted culturally infused PCIT training for clinicians

Findings from the current study underscore the critical need for evidence-based treatment (EBT) developers, including those refining Parent-Child Interaction Therapy (PCIT), to engage diverse clinician voices in shaping culturally responsive clinician training experiences, and organizational implementation efforts. While clinicians consistently value the rigorous stellar training provided in PCIT's core elements, the current qualitative findings suggest specific areas for growth in the evolution of PCIT training methodology, where culturally infused adaptations can significantly enhance the effectiveness, accessibility, and relevance of PCIT for Black clinicians and the Black families they serve.

### 5.1 Differing cultural competency goals for White vs. Black clinician PCIT trainees

#### 5.1.1 Black clinicians: increasing Black representations & leveling the playing field of power imbalance with trainers and the benefits of multiracial cohorts & Black peer mentorship

Effective culturally-infused PCIT training will need to address a dual path of cultural competencies for Black, as well as White clinician trainees, whose needs based on the current study's findings are different. Important future directions for training involves expanding the representation of Black clinicians within the PCIT field, especially as trained PCIT clinicians and in PCIT leadership. Greater visibility of Black clinicians fosters a sense of belonging within the PCIT community, with the potential to enhance recruitment and retention of Black families, as more trained Black clinicians become trusted ambassadors of the model. Leveraging insider cultural knowledge from Black clinicians is vital for tailoring PCIT to better align with the lived experiences of historically marginalized communities.

This also necessitates rethinking mentorship and supervision structures within graduate and clinical training programs for Black PCIT trainees. Attention must be paid to the power differentials Black trainees report experiencing, especially when navigating the dual roles of their PCIT trainers in academic settings. PCIT Training models are encouraged to consider that culturally diverse training cohorts offer a valuable avenue for learning and growth. That is, the increased presence of Black PCIT trainees within cohorts can offer an opportunity for peer mentorship relationships that leverage and affirm the cultural expertise that Black trainees bring to the table. Such incorporation of their insider perspective and knowledge to inform the cultural tailoring of PCIT enables a more leveled playing field of shared power and reciprocal respect in the training paradigm among all parties as suggested in the contact hypothesis proposed by Allport (104) and demonstrated through collaborative exercises outlined by Amir (105). In keeping with the parallel PCIT process of following the trainee's lead within the context of clinician training, such peer-mentorship models would not only support skill development, but also foster collaborative spaces in which clinicians can collectively address challenges such as roadblocks in treatment delivery with Black families. Such multiracially diverse PCIT clinician training teams would also provide the supportive allyship opportunity to challenge notions of traditional hierarchies of power, by allowing Black clinicians to lead, supervise and mentor. This is the same mentorship referenced in the current study, by a White clinician regarding her respected peer mentorship received by her fellow PCIT co-training Black colleague, and as noted by a Black clinician who expressed wanting to receive more mentorship by other Black professionals in positions of PCIT leadership and training.

Chavez et al. (92) and Onovbiona et al. (30) described a Creating Communities Initiative*,* which provided a culturally infused PCIT training adaptation including concordant trainers culturally matching their Black and Latine trainees with a culturally infused PCIT curriculum. Clinicians of color were placed in expert roles in the foreground, while White trainers assumed supportive positions in the background. This shift was well received, offering a respectful space that decentered the Eurocentric nature of PCIT down to its core essence, and prioritized equity and cultural humility. As one clinician noted, it was a “*refreshing experience*” to have space made for leadership from Black voices without the burden of managing White discomfort (30, 92). This subsequently, opened them up to be more receptive to the PCIT model and its potential relevancy for themselves as Black and Latine clinicians, and the Black and Latine families they served.

#### 5.1.2 White clinicians: overcoming reticence, improving cultural competency, and mobilizing allyship

PCIT training should also strive to equip non-Black clinicians with the core cultural competencies, which provide an understanding of these dynamics to be used in the spirit of allyship. That is, using their positional power to support Black families' safety when implementing PCIT publicly to onlookers both within their Black kinship communities, who may seek to undermine newly learned PCIT strategies they do not see as being culturally aligned, as well as onlookers outside the Black community who may pose a threat to the Black child by enlisting public authorities who lack understanding and knowledge about the behavioral intervention.

For White clinicians, PCIT training goals may focus on helping them overcome fears of “*getting it wrong,*” and guiding them toward being allyship-grounded through authentic engagement, rather than through performative gestures, or simple avoidance of addressing such race-related content, as is sometimes evidenced among White clinicians (97), and reported by the current study findings. At the same time, clinicians of color must be supported in navigating complex dynamics where they may share racial identity with clients, but differ in other ways, such as class privilege, which may complicate trust-building. In instances of all clinicians, training PCIT clinicians in how to leverage their individual points of subjugation in their own cultural histories as assessed using Hays' (106) ADDRESSING model, can become points of connection with Black families for trust and rapport building. Building trust remains a central obstacle in racially discordant therapeutic relationships, especially in reference to PCIT, which clinicians described themselves and Black families as being wary of, due to perceptions of it being a predominantly White treatment model.

Obstacles to rapport-building with Black families are often rooted in a healthy and historically justified mistrust of health care systems and providers, stemming from long-standing systemic discrimination. This mistrust can extend to PCIT, which many Black families perceive as “very White” (15). Overcoming these barriers and fostering a strong therapeutic alliance requires clinicians to be intentionally trained to recognize and leverage their own intersectional ethnic identities. Utilizing Hays' (106) ADDRESSING model, clinicians can engage in self-assessment to examine how their identities interact with those of their clients. This process allows for a nuanced understanding of privilege and marginalization on both sides, serving as a bridge-building mechanism from either a place of shared marginalization, or allyships in instances of differential identity power relationships. For White clinicians, in particular, this approach can help reduce their hesitancy, bolster confidence in working with Black PCIT families as cultural outsiders, and transform well-meaning intentions into effective allyship. Importantly, these dyadic therapeutic bridge-building efforts should be complemented by a visible and sustained presence within Black communities to establish credibility. Such engagement helps build trust in racially discordant therapeutic relationships, which can itself be a powerful tool in healing racial trauma.

Likewise, it is critical for Black clients to recognize how perceived disparities—such as differences in socioeconomic status—may also impact the therapeutic relationship, even within a shared racial identity. For example, clinicians who are Black but in positions of perceived power may be seen as having “traded in their race card,” potentially contributing to mistrust or attrition if their therapeutic stance is viewed as upholding White-centered treatment models.

#### 5.1.3 Ecological validation model framework for an adapted culturally-infused PCIT training model

Current research highlights the importance of PCIT training being culturally-infused which is consistent with the APA outlined model of awareness, knowledge, and applications. Future adaptations for a culturally infused PCIT training model can be anchored in established frameworks like Bernal et al.'s (107) *Ecological Validity Model* (EVM), which emphasizes eight cultural domains including language, persons, metaphors, content, goals, methods, and context. This model was applied in the *Creating Communities Initiative* (30, 92), a piloted training program specifically designed for Black and Latine clinicians serving racially diverse families of Autistic youth.

Chavez et al.'s (92) and Onovbiona et al.'s (30) culturally-infused PCIT training model placed PCIT within a Black and Latine centric context, inclusive of core cultural values and concepts germane to Black and Latine ethnocultural theories around parenting through racial socialization. Thereby, addressing critical areas of concerns for Black and Latine caregivers. These involved protection against socio-cultural threats to their children's lives, in tandem with the promotion of healthy cultural identities, cultural pride, and elevated positive self-esteem facilitated by the core foundational PRIDE skills of PCIT as outlined in Chavez's (44) *Tipsheet for applying PCIT principles in servicing Black families and the call to arms insSupport of the BLM Movement* (See Supplementary Appendix C). It discusses how to use the concepts and skills in PCIT, as demonstrated through both the Child Directed Interaction (CDI) PRIDE skills, and Parent Directed Interaction (PDI) to instruct clinicians on how to support Black families in treatment. The training also addressed reparative measures for healing racial discrimination trauma. Adaptations to PCIT's PDI model were also offered regarding potential negative scrutiny of public behavior implementations during the PDI sequence of PCIT. Further, the training incorporated key dimensions from Hays' (106) ADDRESSING model and tailored PCIT to reflect the racial, ethnic, and neurodiverse identities of the families being served.

With a modest sample (*n* = 12) of Black and Latine clinicians, researchers (30, 92) assessed Black and Latine clinician's response to a PCIT training tailored toward Black and Latine clinicians using the culturally-infused PCIT adapted curriculum (44). Following the culturally-infused adapted PCIT training, Black and Latine clinicians largely reported that the PCIT skills could be effectively taught to Black and Latine families (100%), PCIT could be adapted to meet the unique experiences of their Black/Latine clients, the culturally tailored materials provided were helpful for supporting Black and Latine families (87.5%), and they believed that PCIT would be easy to use with Black and Latine families [75%; (92)]. Chavez et al.'s (92) quote from a Black clinician illuminates the sense that they could see the utility and applicability for PCIT within marginalized Black and Latine families in the low-income communities their agency serviced:

“Within our community we see a lot of young moms, and that relationship that's never built, … then kids … make it teenagers, and … they end up getting pregnant, or … leaving home early because they don't have that relationship. So, to find that you could work [on those things] before the issues happen and build that bond between a mom and a daughter, mom and a son, whether they're Hispanic or African American, or of any ethnic background, males it is quite inviting as you project into the future, it makes this [PCIT] training quite significant for our community.”

Lastly, after the training, Black and Latine clinicians perceived themselves as having competence in PCT skills, the latter of which was consistent with the current study data regarding feeling like they received good training with respect to the core rudiments of PCIT. However, after the training, there was less endorsement of PCIT being more effective than other treatments (50%), PCIT's likelihood to produce improvements (37.5%), and PCIT greater convenience in comparison to other therapies (25%). These less favorable endorsements by Black and Latine clinicians were noted following their immediate 40-hour training, and thus for many may have been a function of wanting to see the proof in the pudding following actually carrying families through the treatment process, given some of the other more complex system barriers they were likely to face as common difficulties with their treatment populations that serve as barriers to treatment such as overcoming pre-conceive notions about PCIT.

“Getting the patients in … was really difficult for me. It took me a while to get people to … .trust or ….come in to … just start the treatment.”

As illustrated through the above clinician quote, gaining client trust and starting treatment remained a significant hurdle. These latter findings signaling that while the culturally infused training was perceived as valuable, systemic barriers may still pose challenges to implementation. With respect to other potential barriers, they may be related to feasibility issues with respect transportation, job conflicts, and childcare demands noted in another study (15).

### 5.2 Proposed culturally infused PCIT training curriculum

#### 5.2.1 Cultural content

Training in (PCIT) for clinicians working with Black families must be grounded in culturally-specific curriculum drawn from the lived experiences of Black communities. This approach ensures cultural authenticity, while maintaining treatment fidelity. When delivered from an insider perspective, such as that offered by Black PCIT clinicians who actively tailor treatment to align with cultural values, this content enhances the relevance and effectiveness of PCIT. It also provides a strong foundation for cultural training curricula.

However, clinicians must first understand the historical oppression experienced by Black communities, which has led to a “healthy paranoia*,*” which is a justified skepticism toward health care systems, providers, and research (108). This historical mistrust can present as reticence during intake or early treatment, and if not properly addressed, may contribute to premature dropout among Black families. These same historical elements have shaped Black parenting practices, aligning with what Harkness and Super (31) refer to as *parental ethnotheories.* These are frameworks that guide culturally-grounded parenting goals, particularly around the racial socialization and protection of Black children in racially charged environments, which can result in racial trauma.

Therefore, a culturally adapted PCIT training curriculum should also deepen clinicians' understanding of how transgenerational legacies of racial trauma inform racial socialization parenting practices and traditions, as seen in “*The Talk.*” It is a rite of passage that begins as early as the preschool years, wherein Black caregivers prepare their children for potentially harmful encounters with law enforcement (109). Despite its aim to prepare and protect Black youth it can initially manifest in elevated levels of anxiety, particularly the younger the child. PCIT skills, particularly those focused on emotion regulation, can therefore support caregivers in initiating these critical conversations, using developmentally appropriate language, which balances emotional safety with practical preparation.

Clinicians, especially White allies, should also be trained to use their positional privilege in the spirit of allyship. This includes serving as co-advocates during Parent-Directed Interaction (PDI) public behavior implementations, by anticipating risks, engaging in over-preparation, and offering protection to help families feel secure in public settings.

Racial socialization also involves nurturing children's self-esteem and fostering a strong racial identity as protective factors, which the core essence of PCIT is well-equipped to support through the Child Directed Interaction (CDI) PRIDE skills. However, clinicians must also understand the nature of racial identity development in marginalized populations (43), and how the promotion of racially rooted self-worth leads to the development of Black cultural pride, in order to appropriately tailor and coach PRIDE skill implementation with Black caregivers This is crucial as the development of Black cultural pride in Black youth serves as an emotional and psychological buffer against the harms of discrimination trauma, as well as offering other positive mental health, behavioral, cognitive, and academic benefits (39). Tailoring PCIT's PRIDE skills to align with these Black parental ethno-theoretical goals reinforces their effectiveness for internalization and maintenance.

Language and communication styles are another area in which racial identity and cultural ownership manifest. To affirm these expressions Black cultural pride, adaptations to the Dyadic Parent-Child Interaction Coding System (DPICS) must recognize culturally affirming communication, including the use of African American Vernacular English (AAVE) in the home. Chavez and colleagues (110) emphasize the importance of “Giving Credit Where Credit is Due” in advocating for recognition of AAVE within coding to accurately capture and credit treatment gains achieved by Black caregivers within the 20% degree of latitude for coding reliability.

Finally, training must emphasize culturally responsive assessment practices. This includes gathering meaningful information on parental ethno-theoretical approaches, racial socialization practices, racial identity development, and experiences of discrimination trauma. These insights are essential for tailoring PCIT interventions to the unique needs of Black families and fostering collaborative therapeutic partnerships with Black caregivers grounded in cultural respect and empowerment for Black families (45, 46).

#### 5.2.2 Intake assessments

Clinicians must also be trained to conduct culturally informed intake assessments. This includes recognizing diverse Black family structures as they may unfold in supportive fictive- and non-fictive kinship networks (111). Information regarding kinship supportive networks inform who gets invited to the intake session for a more informed picture of who the key stakeholders are in the child's life, and potentially minimizes any sabotaging of PCIT implementation, especially if such strategies appear on the surface, not to be in alignment with cultural notions of the parental ethnotheories. It will be equally important to address the complex intersections among cultural norms, attitudes regarding corporal punishment with deep seeded historical roots stemming back to slavery as protective interventions for raising their Black children, and discrimination trauma histories. Thus, important assessments of racial socialization practices may inevitably unfold in delicate conversations surrounding the use of disciplinary strategies, and more specifically corporal punishment if used. Nonjudgmental approaches to these conversations are critical for building therapeutic rapport and avoiding the alienation of Black families as early as the intake. Assessments should also explore experiences of racial discrimination and their subsequent psychological impact as it pertains to discrimination trauma for informing the reparative role the PCIT treatment and the therapeutic relationship with the clinician will play, especially in the context of racially discordant providers. Finally, assessments should explore those racial socialization parenting strategies that will serve as protective mechanisms and emotional-psychological buffers in raising Black children. This data will serve as the pivotal crux of PCIT implementation of the CDI PRIDE skills for bolstering self-esteem, and promoting positive healthy racial identity outcomes in the expression of Black PRIDE.

## 6 Conclusion

The cultural curricular resources presented for further enriching PCIT training are rooted in Black cultural experiences. However, many of the strategies—such as supporting families in coping with racial trauma—are transferable to other marginalized communities, including Latine families who also have experienced systemic discrimination, and Asian American populations, who recently at the height of the pandemic experienced significant hate crimes. In sum, future directions for PCIT training must center cultural infusion; not as a supplement, but as an essential component of culturally and ethically sound evidence-based care. Culturally-tailored PCIT training models that reflect and elevate the lived expertise of clinicians of color—while grounding adaptations in established multicultural and ecological frameworks—hold the potential to make PCIT more accessible, effective, and empowering for the communities it aims to serve.

## 7 Limitations

While this study provides many important insights, it also has a few notable limitations. First, the design of the recruitment and interview process leaves room for skewed sampling and relies on the memories and perceptions of clinicians. Logistically, clinician participants were those who volunteered to participate in this research project, and the data was collected via live interviews with clinicians identifiable to the research team. Thus, it is possible that participants were those who had more favorable experiences with Black families or that they may have left out pieces of information that could reflect negatively on their clinical skill with Black families, or people involved in their training with whom they may have continuing relationships. Second, while clinician perspectives are a valuable and needed addition to the literature, clinicians' representations of their work with Black families may not accurately reflect the families' experiences of working with the clinician. Thus, diversity is needed in reporting on Black familial consumer perspectives as well. Third, we used an adapted version of a PCC measure that was originally developed for primary care medical providers. Our adapted version has not been validated, and certain items may lack relevance to the study population. However, we consider it a strength that we were able to assess perceived PCC at the provider level in this study. Lastly, this participant sample was racially diverse, however, it did not represent the full spectrum of possible races and ethnicities with the notable absence of Latine and Native American providers among others where representation was fully absent or perhaps could have benefitted from higher numbers of participants. Given these absences, more research should be done to include more voices of racially and ethnically diverse clinicians. Further, future researchers would do well to ask about diversity across other domains (i.e., ability status, immigration status, etc.) and intentionally recruit participant samples that represent wide swatches of the diverse clinical workforce that is required to meet the needs of all the families we serve.

## Data Availability

The original contributions presented in the study are included in the article/Supplementary Material, further inquiries can be directed to the corresponding authors.
